# Patient-derived tumor xenograft and organoid models established from resected pancreatic, duodenal and biliary cancers

**DOI:** 10.1038/s41598-021-90049-1

**Published:** 2021-05-19

**Authors:** Nhu-An Pham, Nikolina Radulovich, Emin Ibrahimov, Sebastiao N. Martins-Filho, Quan Li, Melania Pintilie, Jessica Weiss, Vibha Raghavan, Michael Cabanero, Robert E. Denroche, Julie M. Wilson, Cristiane Metran-Nascente, Ayelet Borgida, Shawn Hutchinson, Anna Dodd, Michael Begora, Dianne Chadwick, Stefano Serra, Jennifer J. Knox, Steven Gallinger, David W. Hedley, Lakshmi Muthuswamy, Ming-Sound Tsao

**Affiliations:** 1 Princess Margaret Cancer Centre, University Health Network, Toronto, ON Canada; 2 Ontario Institute of Cancer Research (OICR), Toronto, ON Canada; 3 Lunenfeld-Tanenbaum Research Institute, Mount Sinai Hospital, Toronto, ON Canada; 4 Division of Medical Oncology, Princess Margaret Cancer Centre, University Health Network, Toronto, ON Canada; 5 Department of Pathology, UHN Program in BioSpecimen Sciences, University Health Network, Toronto, ON Canada; 6 Department of Laboratory Medicine and Pathobiology, University of Toronto, Toronto, ON Canada; 7 Division of General Surgery, University of Toronto, Toronto, ON Canada; 8 Cancer Center, Beth Israel Deaconess Medical Center, Harvard Medical School, Boston, MA USA

**Keywords:** Cancer models, Biliary tract cancer, Pancreatic cancer, Cancer, Oncology

## Abstract

Patient-derived xenograft (PDX) and their xenograft-derived organoid (XDO) models that recapitulate the genotypic and phenotypic landscape of patient cancers could help to advance research and lead to improved clinical management. PDX models were established from 276 pancreato-duodenal and biliary cancer resections. Initial, passage 0 (P0) engraftment rates were 59% (118/199) for pancreatic, 86% (25/29) for duodenal, and 35% (17/48) for biliary ductal tumors. Pancreatic ductal adenocarcinoma (PDAC), had a P0 engraftment rate of 62% (105/169). *KRAS* mutant and wild-type PDAC models were molecularly profiled, and XDO models were generated to perform initial drug response evaluations. Subsets of PDAC PDX models showed global copy number variants and gene expression profiles that were retained with serial passaging, and they showed a spectrum of somatic mutations represented in patient tumors. PDAC XDO models were established, with a success rate of 71% (10/14). Pathway activation of KRAS-MAPK in PDXs was independent of *KRAS* mutational status. Four wild-type *KRAS* models were characterized by one with *EGFR* (L747-P753 del), two with *BRAF* alterations (N486_P490del or V600E), and one with triple negative *KRAS/EGFR/BRAF*. Model OCIP256, characterized by *BRAF* (N486-P490 del), had activated phospho-ERK. A combination treatment of a pan-RAF inhibitor (LY3009120) and a MEK inhibitor (trametinib) effectively suppressed phospho-ERK and inhibited growth of OCIP256 XDO and PDX models. PDAC/duodenal adenocarcinoma have high success rates forming PDX/organoid and retaining their phenotypic and genotypic features. These models may be effective tools to evaluate novel drug combination therapies.

## Introduction

Tumors from the periampullary region include pancreatic, ampullary duodenal and distal bile duct cancers. Malignancies from these different organs are associated with distinct biological behavior and mortality rates. The 5-year overall relative survival rate for pancreatic cancers is 10%, bile duct cancers is 19.6%, and ampullary duodenal cancers have a 5-year survival rate of 60%^1,2^. The anatomical proximity of these cancers contributes to their similar clinical presentation and therapeutic options. Pancreato-duodenal and biliary cancers are diagnosed often at advanced disease stages with intestinal or bile duct obstructive symptoms and tend to respond poorly to systemic therapies. The molecular landscapes of these cancers show diverse abnormalities in sustained tumor growth, cell cycle and cell signaling pathways that may influence disease resistance to systemic and targeted therapies^3–5^. There are no validated biomarkers to assess individual tumor susceptibility to such targeted or systemic therapies in these cancers besides targeting DNA repair factors with poly-ADP ribose polymerase inhibitors in Pancreatic Ductal Adenocarcinoma (PDAC) harboring germline *BRCA* mutations^6–9^.

PDAC is the most common and widely characterized periampullary cancer. The majority (> 90%) of these tumors harbor the oncogene Kirsten RAt Sarcoma virus mutations (*KRAS*^mt^), complex chromosomal rearrangements involving chromothripsis and copy number changes^10,11^. *KRAS* oncogene is a potent activator of Mitogen Activated Protein Kinase (MAPK) pathway driving PDAC initiation and progression. Despite intensive efforts, current therapeutic advances in this disease is extremely limited^9^. A minority of PDACs has *KRAS* wild-type (*KRAS*^wt^), but may also exhibit MAPK deregulation via somatic alterations in genes activating Receptor Tyrosine Kinase (RTK) signaling in RTK/RAS/MAPK cascade. Additional pathway mediators that are altered may include *BRAF* in-frame deletions (~ 1%), *NRG1* gene fusions (< 1% to 11%) and *ERBB2/HER2* amplifications (2–24%)^12–16^. Targeted therapies to inhibit RTKs, ERBB1/EGFR (e.g. erlotinib) and/or ERBB2 (e.g. afatinib or pertuzumab) in patients with *KRAS*^wt^ and *NRG1* fusion PDAC, have resulted in promising initial responses^14^. New therapies in bile duct and periampullary adenocarcinomas are also not forthcoming^17^. Therefore, new clinically relevant models for these cancers are needed to better understand their biology and for discovery of new therapeutics against these cancers evading treatment.

While few hundreds PDAC PDX models have established worldwide, few studies with limited number of models have reported detailed characterization of their establishment, phenotypic characterization and molecular profiling^18–24^. Similarly, for PDX models of bile duct cancers^25,26^. Patient tumors and PDX may also be used to establish patient-derived organoid (PDO) models, and these latter models are useful for initial higher throughput functional genomic studies, with subsequent validation in the matched PDX models^27^. Establishing a renewable PDX/PDO resource with relevant clinical annotation, and genotyping is essential for preclinical studies in new drug and biomarker discoveries^11,20^. In this manuscript, we described our initiative to establish 127 PDX from resected pancreato-duodenal and biliary cancers and 10 matching PDAC xenograft-derived organoid (XDO) models. A subset of PDAC was further molecularly profiled for genomic fidelity to corresponding patients by genome-wide copy number and gene expression. In addition, whole exome profiling was performed on 31 PDAC PDXs to identify potential drug actionable alterations. Using a subset of *KRAS*^*wt/mt*^ PDAC models, we showed that these PDAC tumors exhibited heterogeneous KRAS-MAPK pathway activation which can be targeted using a rational polytherapy approach in patient-derived organoid and their xenograft models.

## Results

### PDX model establishment from pancreatic-duodenal-biliary system

**Figure 1:**
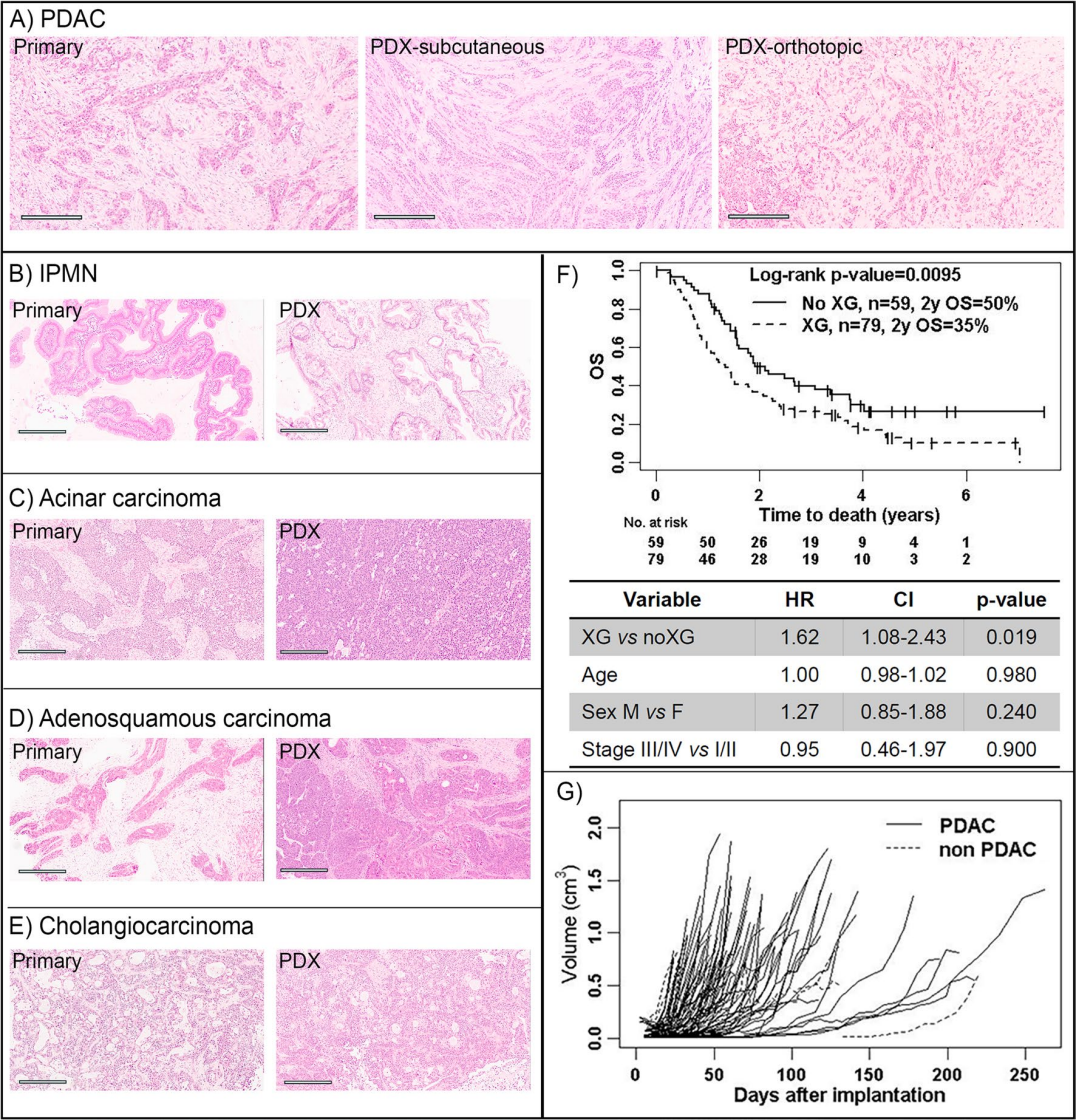
Establishment of PDX models from resected tumors. Pairs of patient primary-PDX tumors show a high overlap of histological features. (**A**) A poorly differentiated PDAC (OCIP88) with cords of infiltrating tumor cells. (**B**) An IPMN (OCIP250) with papillary growth lined by columnar and mucinous tumor cells. (**C**) An acinar cell carcinoma (OCIP270) showing tumor cells with eosinophilic cytoplasm growing in sheets. (**D**) An adenosquamous carcinoma (OCIP130) showing highly similar histological appearances in both the primary and PDX tumors. (**E**) A cholangiocarcinoma (OCIP194) with focal glandular structures. Scale bars are 300 μm. Histology images were color balanced to compensate for over-/under-staining, no features were altered in the process. (**F**) A significantly lower rate of overall survival (OS) was associated with patients’ tumors that had successful initial engraftment (XG at P0) compared to those that failed engraftment (No XG). A multivariate table shows analysis of variables with hazard ratio (HR). (**G**) Growths of individual pancreatic tumors to an average of 1.5 cm^3^ at humane endpoint, at the flank of NOD SCIDs.

Between September 2008 and June 2013, a total of 276 Whipple resected samples were collected for PDX establishment from pancreas (n = 199), duodenum/ampulla of Vater (n = 29), and extrahepatic bile duct (n = 48), as summarized by histological subtypes (Table 1), and inventory tables (Supplementary Tables S1 and S2). The PDX tumors retained the morphological features of their matched patient tumors, as shown in these representative cases, Fig. 1, A-E. Both orthotopic and subcutaneous sites showed fidelity of the poorly differentiated PDAC features to their matched patient tumors (Fig. 1A). Compared to the patient tumors, the PDX tumors at either the orthotopic or subcutaneous site showed a tendency toward enrichment of tumor cells compared to stromal components. We did not observe metastases at either the subcutaneous or orthotopic tumor growths, although in the latter situation, larger tumors may invade into adjacent organs, such as intestines, spleen and liver.

**Table 1:** Number of implanted specimens and engrafted xenograft tumor models.

Primary Site	Patients	No-XG (%)	XG_P0 (%)	Stable XG (%)
**Pancreas**	**199**	**81 (41)**	**118 (59)**	**99 (50)**
PDAC*	169	64 (38)	105 (62)	89 (52)
IPMN**	15	10 (66)	5(33)	3 (20)
Mucinous adenocarcinoma	4	1 (25)	3 (75)	3 (75)
Squamous carcinoma	1	0 (0)	1 (100)	1 (100)
Undifferentiated carcinoma	2	0 (0)	2 (100)	1 (50)
Solid pseudopapillary carcinoma	2	2 (100)	0 (0)	0 (0)
Acinar cell carcinoma	1	0 (0)	1 (100)	1 (100)
Neuroendocrine carcinoma	5	4 (80)	1 (20)	1 (20)
**Biliary duct**	**48**	**31 (64)**	**17 (35)**	**8 (17)**
**Ampullary-duodenum**	**29**	**4 (14)**	**25 (86)**	**20 (69)**
**Total**	**276**	**116 (42)**	**160(58)**	**127(46)**

Patient-derived tumor models (XG) after initial passage (P0) in mice and serial passages for at least three passages to evaluate stable xenograft propagation (SXG). *PDAC* pancreatic ductal adenocarcinoma; *IPMN* intraductal papillary mucinous neoplasm.

*A subset of PDAC were ascites specimens; 6/21 had initial engraftment and also formed SXG models.

**15 IPMNs included eight with invasive adenocarcinoma; XG_P0 IMPN cases were associated with invasive adenocarcinoma (4/5) and one with focal invasion; Stable XG IPMN cases were associated with invasive adenocarcinoma (n = 2), and focal invasion (n = 1).

Engraftment rates at the subcutaneous pocket at the flank of NOD SCID mice varied across cancer types. Ampullary-duodenal tumors had 86% (25/29) initial engraftment rate (P0), and 69% (20/29) as stable xenograft (SXG) models (Table 1). Bile duct tumors showed success rates of 35% (17/48) at P0 and 17% (8/48) at SXG. Pancreatic tumors had an overall success rate of 59% (118/199) at P0, and most could be propagated serially as SXG (50%, 99/199).

Among the pancreatic tumors, PDACs showed the highest initial patient to mouse engraftment rate (62%, 105/169), compared to the combined non-ductal tumors at (53%, 8/15), and IPMNs (33%, 5/15). Among the 15 IPMNs, eight were associated with invasive adenocarcinoma; these tumors demonstrated initial engraftment rate (P0) of 50% (4/8). For the remaining seven IPMNs, only one (14%) engrafted (P0). Retrospective review of the surgical pathology slides of the latter case demonstrated severe dysplasia with areas of focal invasion (Supplementary Fig. S1A).

Additional to the subcutaneous site, PDAC patient tumor fragments were implanted at the orthotopic site (Supplementary Table S3), with an engraftment rate of 22% (16/71), as compared to 100% (71/71) when implanted subcutaneously. Among the 169 PDAC tumors, the ability to engraft at the subcutaneous site was not associated with specific clinical pathological features (Supplementary Table S4). However, engraftment was significantly correlated with poorer patient overall survival (Fig. 1F, p-value = 0.0095), but not recurrence rate (Supplementary Fig. S1B).

### Growth characteristics of PDAC xenografts

A subset of PDAC models was further characterized by xenograft tumor latency (n = 34) and growth rates (n = 23). The latency time between tumor implant and palpable detection was observed for more than one mouse per model per passage, with a median of 18 days after initial implantation (P0), and serial passages resulted in shorter but stable median latency times ranging 11–14 days (Supplementary Fig. S1C). At passage 4 all had palpable tumors within 46 days (Supplementary Table S5A). Most (90%) of the models had P0 palpable tumors within 90 days (Supplementary Table S5B). Latency at P0 or P1 did not correlate with patient overall survival (Supplementary Fig. S1D,E). However, a tendency for a correlation of shorter latency times in P1 with early recurrences was observed in this small subset of PDAC models (Supplementary Fig. S1F,G).

Tumor growth rates in P1 varied among models of the different pancreatic tumors (Fig. 1G), and for duodenal and bile cancers (Supplementary Fig. S2A–D). Tumor models derived from the duodenum grew significantly faster than the pancreas (p-value < 0.0001, Supplementary Fig. S2E). Since there were only two bile duct PDX models, their growth rates were not analyzable. Tumor latency duration was not associated with tumor cellularity (Supplementary Fig. S2F).The growth rates in the PDAC subset was not significantly associated with clinical outcome (Supplementary Fig S3A,B).

### Molecular profiling of PDAC models

Copy number and gene expression profiling was performed on 4 randomly selected PDX models to determine the genomic fidelity among serial passages (P0-P4). The majority of the gene copy number changes observed in early passages were retained overtime as profiles clustered within a model (Supplementary Fig. S4A). Gene expression profiles showed high concordance across passages within a model (R > 0.97, Supplementary Fig. S4B).

*KRAS* mutations by direct sequencing were detected in 95% (73/77) of the PDAC models. However, four (5%) PDAC PDX and their matched patient tumors were *KRAS*^wt^. Whole exome sequencing was performed on 31 PDX models, including the 4 *KRAS*^wt^, of which 23 PDXs had corresponding patient tumor profiles. The PDX cohort had a median of 63 somatic mutations compared to 49 mutations in the patient cohort. The median proportion of patient tumors’ somatic mutations that were found in their matched. PDX models was 76% (range 18–95%); 21/23 cases had > 55% representation except for 2 cases with 18% and 30% overlaps (Supplementary Table S6). Vice versa, median proportion of PDX somatic mutations that were found in their matched patient was 53% (range 1–94%).

**Figure 2:**
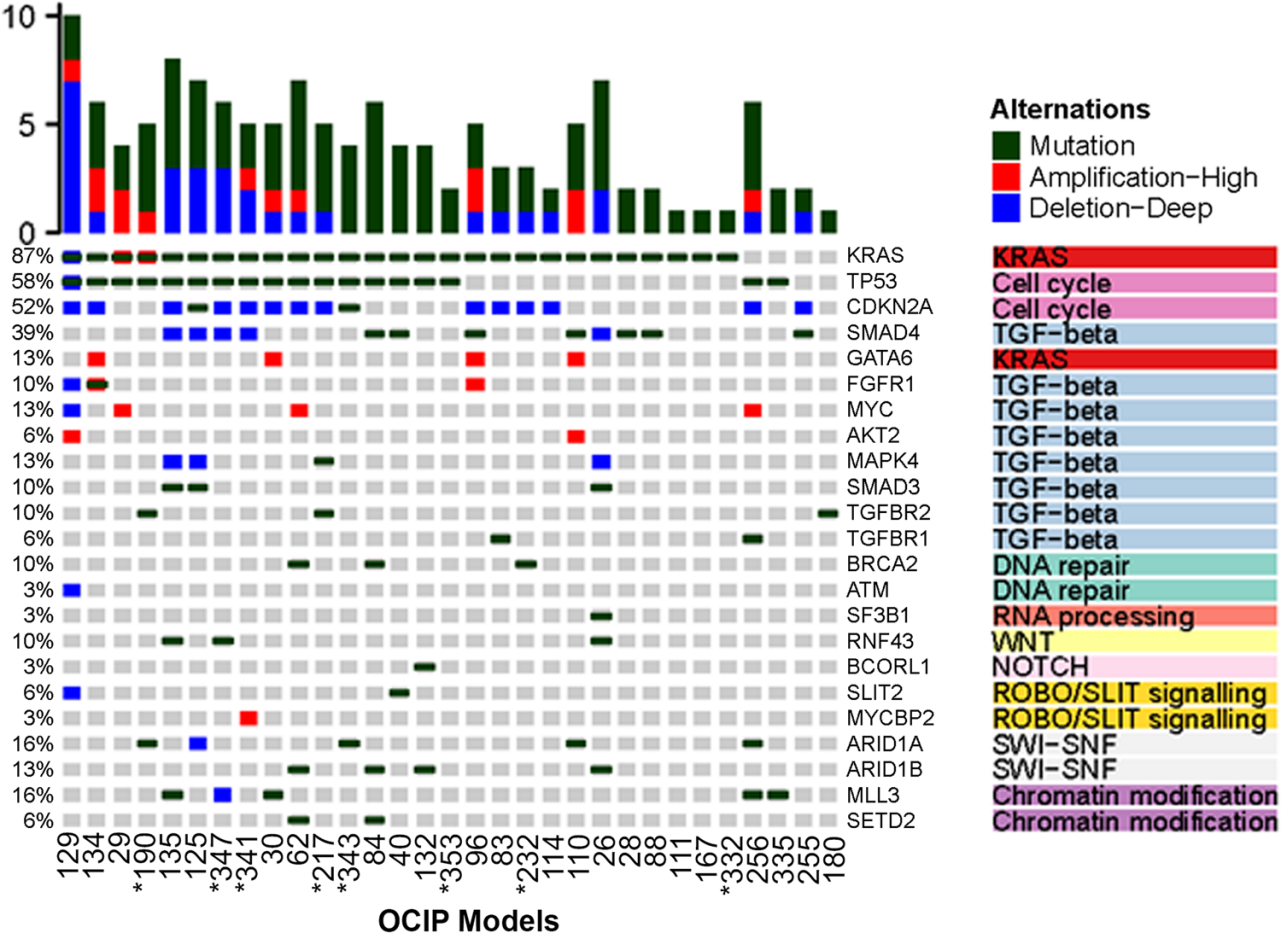
Somatic mutations in PDAC PDX models. Frequently mutated genes in PDAC are represented. Whole exome profiles were used for analysis, and * cases indicate whole genome profiles.

The somatic profile on 31 PDX models were characterized by alterations typically found in the PDAC landscape (Fig. 2), and included the common PDAC-associated genomic aberrations such as *TP53, SMAD4, CDKN2A, BRCA1/2*^13^, and 18 of the 32 genes that were significantly mutated in a 456 patient-set reported by Bailey et al.^10^. Somatic with or without germline or germline alone mutations in *BRCA1/2* that lead to homologous recombinant deficiency were found in 19% (6/31) models profiled (Supplementary Table S7).

### Establishment of organoid lines from PDX models

**Figure 3:**
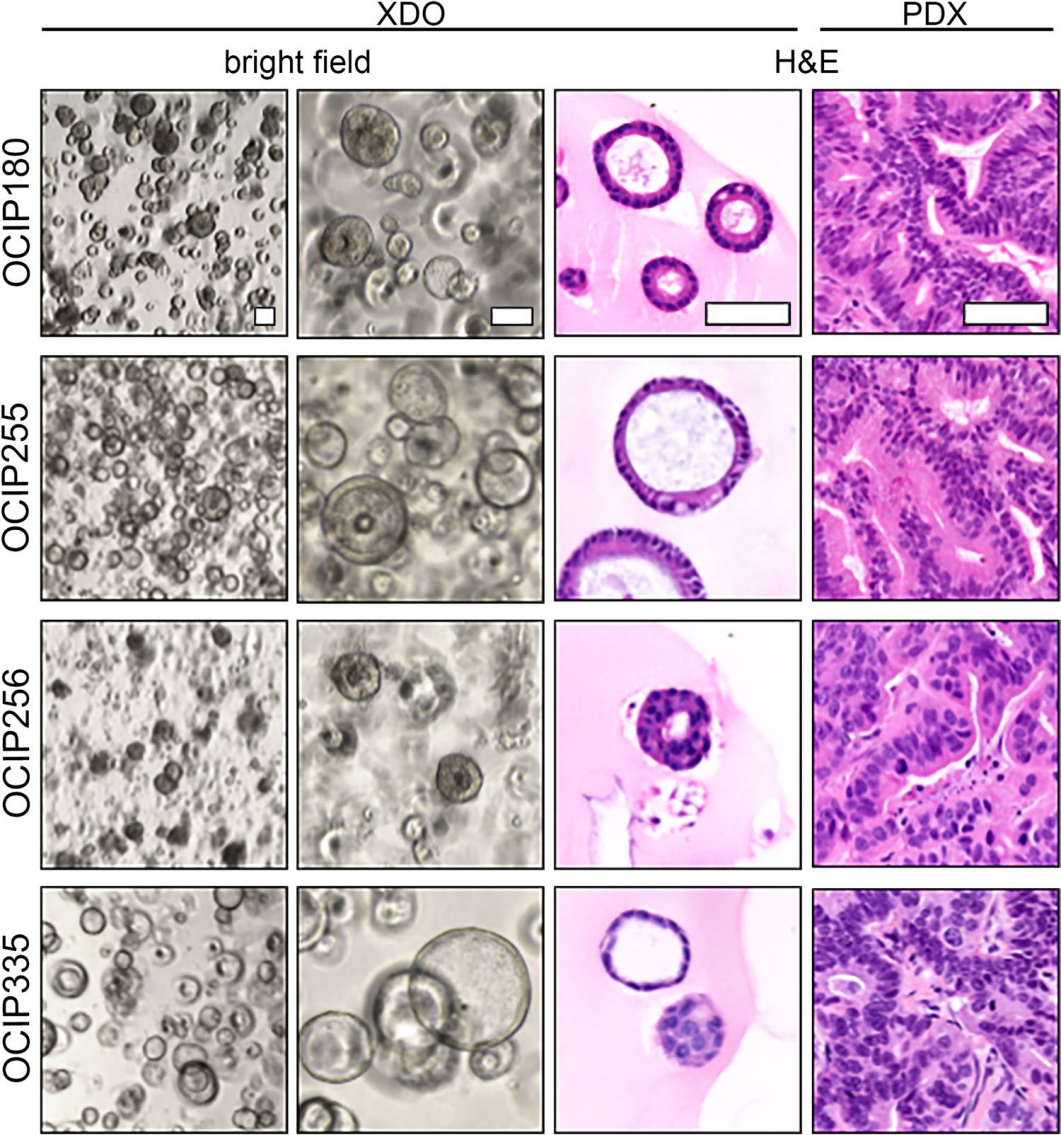
Bright field and histology images of XDO models and their parent PDX models. Scale bars are 100 µm.

Xenograft-derived organoid (XDO) lines were successfully established from 10/14 (71%) of the PDAC PDX models. These models could be maintained long-term (> 5) passages. Histological fidelity between the parent xenografts and derived organoids was preserved as shown with representative models (Fig. 3).

### Drug sensitivity profiling in matched XDO and PDX models

**Figure 4:**
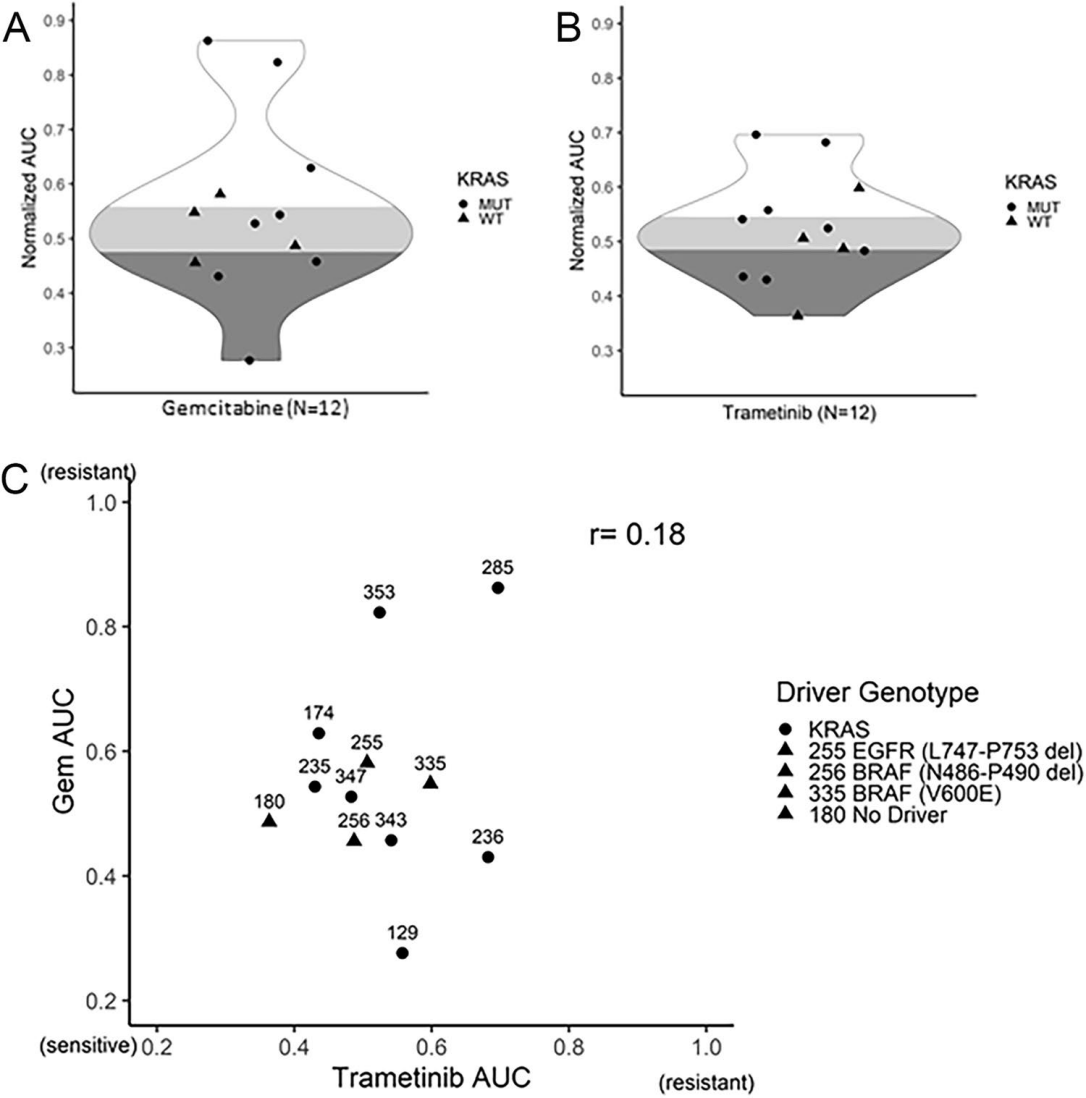
Drug dose responses of XDO models. (**A**) Gemcitabine and (**B**) trametinib cell viability scores were calculated by area under the curve (AUC) for *KRAS* mutant and wild-type models. Cell viability was measured using a Celltiter Glo 3D assay. (**C**) The scatter plot of organoid AUC scores were compared for both drugs. Model OCIP180 was identified as No Driver (triple negative *KRAS*/*BRAF/EGFR*).

Ten XDO models were tested for sensitivity to gemcitabine, a standard of care chemotherapy for PDAC patients, and trametinib, an inhibitor of MEK1/2. These models included 6 *KRAS* mutants (*KRAS*^mt^ 3 G12D, 2 Q61H, 1 Q61L), and 4 *KRAS* wild-type (*KRAS*^wt^), the latter included 2 *BRAF* mutants (V600E and N486_P490del), one *EGFR*^L747_P753del^, and one “no driver” negative triple mutant *KRAS/EGFR/BRAF*. The *KRAS*^mt/wt^ models had non-distinct sensitivities towards gemcitabine or trametinib (Fig. 4A,B and Supplementary Fig. S5A,B). Drug sensitivities were not correlated with each other (Fig. 4C), and were not associated with organoid cell doubling time (Supplementary Fig. S5C,D). In the *KRAS*^wt^ XDOs (OCIP255, -256, -335, -180), the partial response to trametinib suggested that MAPK signaling may still contribute to regulating the growth of the tumor cells (Fig. 4B).

**Figure 5:**
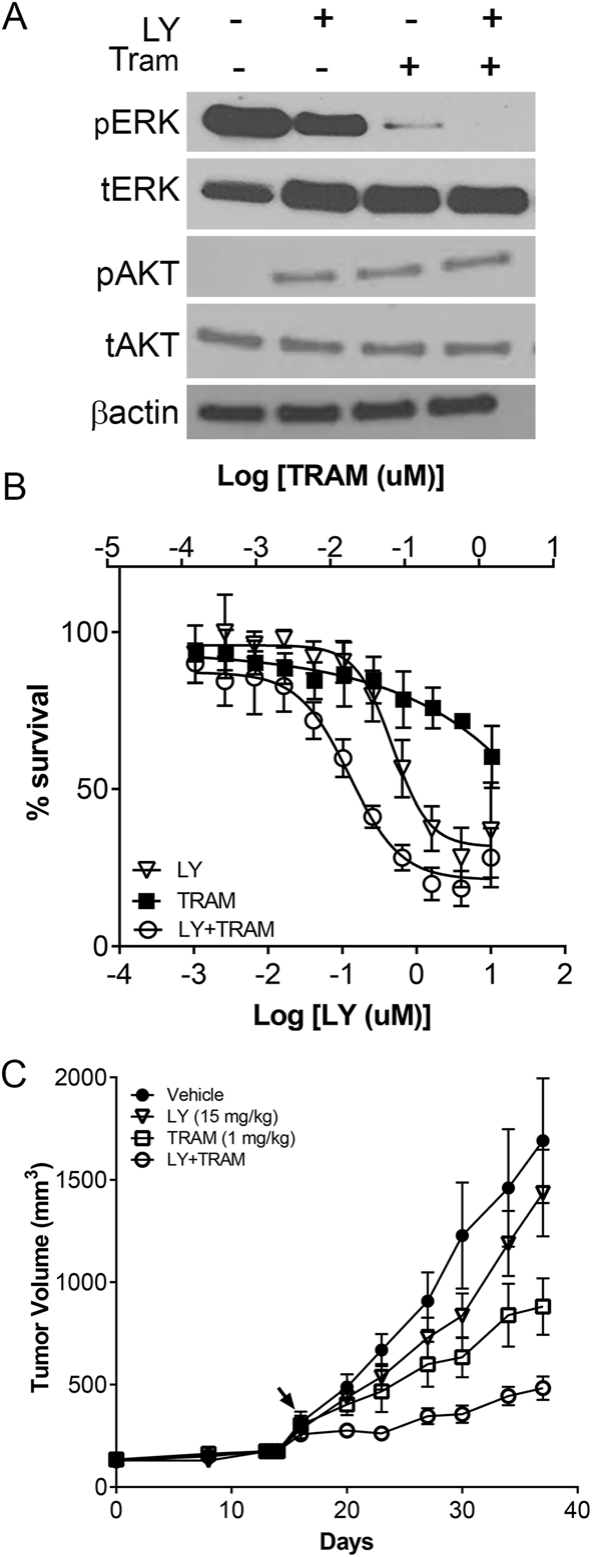
Organoid OCIP256 with *KRAS*^wt^ and putative actionable *BRAF* had activated phospho-ERK (pERK) and phospho-AKT (pAKT). (**A**) Levels of pERK decreased with either LY3009120 (LY) and/or trametinib (Tram) treatments. Original scanned films are shown in Supplementary Fig. S8. (**B**) Inhibition of XDO tumor cell growth was measured with drug treatments, LY3009120 (10–0.001 uM), trametinib (1.25–0.0001 uM), or combined LY:Tram at 8:1ratio. (**C**) Drug sensitivities were evaluated in its parent PDX model.

Model XDO 256 *BRAF*^N486_P490del^ was further assessed for sensitivities to trametinib and a pan-BRAF inhibitor LY3009120, alone or in combination. Western blots showed partial inhibition of pERK by trametinib or LY3009120 alone, and complete inhibition when they were combined (Fig. 5A). This greater inhibition signaling inhibition was also associated with greater growth inhibition in XDO tumor cells when treated with drug combination compared to single agent treatment (Fig. 5B).

The corresponding PDX models also showed that the KRAS-MAPK pathway activation occured independent of *KRAS* genotype as shown by the phospho-ERK and phospho-AKT levels (Supplementary Fig. S6). PDX models, which included one *KRAS*^Q61R^ (OCIP285) and one triple negative, wild-type *KRAS*/*EGFR/BRAF* (OCIP180) were both significantly growth suppressed when treated with trametinib, with the *KRAS*^*mt*^ model showing greater response than the *KRAS*^wt^ triple negative model (Supplementary Fig. S7). As directed by the molecular and phospho-ERK profiles, targeted drug combination treatment with trametinib and LY3009120 showed greater inhibition in PDX OCIP256 (*BRAF*^N486_P490del^) as compared to single agent treatments, mirroring the finding in XDO (Fig. 5B,C).

## Discussion

We attempted engraftment of 276 resected pancreato-duodenal and biliary cancers patient tumor specimens in immunodeficient mice to generate PDX models. The initial engraftment rate at P0 was highest for duodenum at 86% (25/29), followed by 59% (118/199) for pancreatic tumors, and 35% (17/48) for bile duct adenocarcinoma. Among the 160 initial engrafters, 127 PDXs formed stable models (99 pancreatic, 20 duodenal and 8 bile duct), which were sustainable for at least three serial passages. In four PDAC PDX models, we showed stability of copy number and gene expression profiles up to 4 passages. Whole exome sequencing and copy number profiling of 31 models revealed similar landscape of genomic changes as observed in patient tumors^10,13,28,29^. In addition, organoid lines were successfully established from 10/14 (71%) of PDAC PDXs, a subset of which were subjected to drug testing.

Our establishment of PDX models from pancreatic-biliary-duodenal cancers represent the largest effort published by individual institutions. A review of published literatures showed a combined total of 114 pancreatic, and 54 bile duct PDX models (Supplementary Table S8), few of them have had full details on engraftment stability, genome-wide molecular profiling completed, and made publicly available. In addition, the PDXFinder data repository (pdxfinder.org) revealed additional models that may be available from various consortium and commercial sources. The 20 ampullary duodenal carcinoma PDXs we are reporting represent the largest collection known. The establishment rate for duodenal carcinomas is high at 86% initial and 69% stable engraftment. In addition to 54 bile duct (cholangio) carcinoma PDXs that have putatively been established, we generated an additional 8 stable PDXs, with initial (P0) success rate of 35%, which dropped to 17% for stable models. From the study reporting largest number of bile duct PDX establishment, the engraftment rate was independent of corresponding patients’ tumor stage and overall survival, but was significantly associated with a shorter time to recurrence. Due to our small collection of bile duct cancers, engraftment and clinic-pathology correlations were not feasible.

We generated a total of 99 pancreatic PDX models, including 97 carcinomas of duct epithelial origin, 1 acinar carcinoma and 1 neuroendocrine carcinoma. The duct epithelial origin carcinomas included 92 ductal, 3 mucinous, 1 squamous and 1 undifferentiated. Furthermore, 3 of the ductal carcinomas were established from IPMN tumor fragments. The majority (80%) of the IPMN specimens failed to form stable PDX. This is consistent with IPMN being mainly *in-situ* neoplastic proliferation. The three IPMN-associated stable PDXs had corresponding patient tumor with invasive adenocarcinoma or severe dysplasia with focal invasion.

Multiple groups have reported establishment of PDX models from PDAC with engraftment successes ranging from 43% (57 PDXs)^30^ to 85% (35 PDXs)^31^, (Supplementary Table S8). Our success rates are comparable for initial (62%) and stable (52%) engraftment. A large factor that influenced engraftment success appears to be tumor cellularity and quantity of the implanted fragment. Studies reporting higher success rates used larger implanted fragment (5 mm^3^)^31^ compared to smaller fragment sizes (< 3 mm^3^) in our study, and others^30^. In our study, lowest engraftment rates were observed for orthotopic implant sites (16/71, 22%), and implant sources from ascites specimens (6/21, 28%), and was likely due to smaller size of tumor tissue implanted in the orthotopic method, and fewer tumor cells in ascites specimens, respectively. Loukopoulos et al*.* and Perez-Torras et al.^32,33^ have suggested that one advantage of the orthotopic compared to subcutaneous implantation has been its metastatic potential to distant organs, otherwise they have been shown to have similar global gene expression^22^. However, we observed that tumors implanted in the pancreas could invade into adjacent organs, such as intestines, spleen, liver etc., when they grow larger, mimicking locally invasive pancreatic cancer in patients (results not shown). The use of mouse strains less immunocompromised than the NOD SCID may impact to lower engraftment rates. For example, the Nude strain yielded a 43% engraftment success (absent thymus and T cells)^30^ compared to our set (62% at P0). However, the Nude strain was also reported to yield a high engraftment rate of 71% from ascites (Supplementary Table S8). These observations suggest multiple conditions favoring engraftment, a more immunocompromised mouse strain, aggressive tumor characteristics and high tumor cellularity/larger implant fragments. Specific to our method, Matrigel® may have supported the initial tumor engraftment.

Tumor growth characteristics of the PDAC models did not significantly associate with specific clinicopathological features of the patient tumors. However, successful tumor engraftment was highly correlated with poorer overall survival of the patients. This has been a consistent observation across different tumor types including non-small cell lung, head and neck and bile duct cancers^34–36^.

Our evaluation of global copy number and gene expression through early serial passages (P0-P4) indicated that the global similarities were retained overtime in a representative four PDAC PDX models. This is consistent with other studies using few representative models to show genomics fidelity between PDX early and late passages, as well as to their matched patient tumors^20,22^, and between primary, metastasis and PDX^24^.

Whole exome sequencing on paired PDAC patient tumors and PDXs showed high fidelity in their somatic mutation profile. Globally, some discrepancies in somatic mutations were observed between matched patient-PDX tumors. Several factors may contribute to these discrepancies, in particular specific to our dataset, existing data was derived from either whole genome or exome sequencing methods which may have variant detection biases (e.g. target regions, depth of coverage). Additional contributors, also reported by other studies, include tumor enrichment observed in PDX compared to their matched patients^22^, and the incomplete removal of mouse variants orthologous to human genes^24^. Consistent with these reported PDX-patient tumor evaluations, our results also showed that PDAC-associated mutations were represented in PDXs. Excluding *BRCA1/2,* these somatic mutations are not currently drug targetable.

To increase the utility of PDX models, we successfully generated 10 xenograft-derived organoids (XDO) with a 71% rate of success. Active MAPK pathway signaling as profiled by phospho-ERK and phospho-AKT were observed in both wild-type and mutant *KRAS* models. XDO drug screens followed by PDX validation, showed that these models had some sensitivity to trametinib regardless of the *KRAS* status suggesting that MAPK signaling is a core proliferative signaling pathway in PDAC. One of the *KRAS*^*wt*^ models, OCIP256 also has an oncogenic in-frame *BRAF* deletion (N486-P490del). This alteration was previously targeted by a RAF dimer inhibitor LY3009120 in BxPC3 PDAC cell line^37^. Interestingly, XDO/PDX OCIP256 tumor growth was more suppressed with trametinib treatment compared to LY300920 alone (Fig. 5). Our data is corroborated with the recent clinical evidence that oncogenic in-frame *BRAF* deletions confer sensitivity to MAPK signaling^12^. While one patient carrying *BRAF* in-frame deletion had a partial response to a MEK1/2 inhibitor, the other patient showed no response highlighting a need to identify effective combination therapy strategies with MAPK inhibition. We confirmed the synergistic effect of the LY3009120/trametinib combination in OCIP256 PDO and XDO models (Fig. 5). To our knowledge, the OCIP256 model is the first patient-derived xenograft and organoid model derived from a PDAC patient with *BRAF* in-frame deletion and could be a useful model to investigate effective combination strategies with MAPK inhibition.

The transition between these preclinical platforms would be cost effective for studies that could perform high-throughput drug treatment strategies, followed by validation of the fewer conditions using more complex in vivo PDX environment testing. These pancreas PDX models which are clinically annotated and capture the genetic diversity of the patients will be used as a preclinical tool to better understand the biology of pancreatic cancers and its underlying impact on effectiveness of therapeutic approaches. These PDX and XDO models are an invaluable renewable tumor resource for research.

## Methods

### PDX establishment

The University Health Network (UHN) Human Research Ethics and Animal Care Committees approved this study protocol (REB# 08-0767 T). Surgical and ascites specimens were collected at the Toronto General Hospital (TGH-UHN) between September 2008 and June 2013 with informed consent from participants. Human research followed the guidelines of Canada Tri-Council Policy Statement, in accordance with Declaration of Helsinki (www.pre.ethics.gc.ca.). Tumor specimens were collected in serum-free Dulbecco’s Modified Eagle’s Medium (DMEM), and used within 24 h. Fragments (4–8 mm), or cell pellets were mixed with 10% Matrigel™ (Product Number 354234, Corning) at 4 °C, and implanted in the subcutaneous pocket at flank, or orthotopic site in Non-Obese Diabetic Severe Combined Immune-Deficient (NOD SCID) mice^38^. Animal care followed the guidelines of UHN Research Institutes’ policies and the guidelines of the Canadian Council on Animal Care, and consistent with ARRIVE guidelines for study design^39^.

Tumor growth was measured twice weekly with a caliper for the length (L, largest diameter) and its perpendicular width (W), including skin fold. The volume was calculated using the formula V = W^2^ × L/2. Tumor latency/lag phase was recorded and defined as the duration between tumor implant to first palpable tumor detection. The initial implant of patient tumor fragment into mouse host was defined as passage 0 (P0), followed by serial propagation of tumor fragments in subsequent new hosts. Tumors were harvested once they reached a humane endpoint size of 1.5 cm in largest diameter, divided for for further studies, and cryo-banking at the Princess Margaret Living Biobank (PMLB). Engraftment (XG) was successful if tumors established in the initial tumor implant (P0), and as stable engrafters (SXGs) in at least another 2 successive passages. Concordance of paired patient-PDX samples were compared by histology hematoxylin and eosin (HE) stained sections, and by DNA fingerprinting (AmpFLSTR™ Identifiler™). The PDX Metadata is available at PDX Finder web-based data portal^40^. Whole slides of Haemotoxylin and Eosin (H&E) histology sections were digitally scanned with 20 × objective (Aperio AT2 brightfield scanner, Leica Biosystems Inc., Buffalo Grove, IL, USA). Western Blot analyses were performed using primary antibodies phospho-ERK (T202/Y204; #9101), total ERK (#9102), phospho-AKT (S473; #9271), and total AKT (#9272) was obtained from Cell Signaling Technology Inc (Danvers, MA, USA), and β-Actin antibody (#A1978, Sigma-Aldrich Corp. St.Louis, MO, USA). Membranes were probed with secondary anti-rabbit/mouse IgG, HRP-linked antibodies (#7074 and #7076; Cell Signaling Technology) for 1 h prior to imaging. ECL reagent (GE Healthcare, Chicago, IL, USA) was used to detect proteins of interest as previously described^41^.

### Genomics profiling

Whole exome sequencing was performed using samples prepared with the Agilent SureSelect Human All Exon V4 capture kit. Next generation sequencing for whole exome and whole genome sequencing was performed using Illumina HiSeq 2000/2500 instruments on paired-end libraries at the Ontario Cancer Research Institute (Toronto, ON, Canada). Data analysis protocol was based on previously described methods^42^. Illumina’s CASAVA software (version 1.8.2) converted the sequencing base calls to fastq format reads, after which, Xenome^43^ was used to remove any mouse contamination reads. Reads were aligned to the human reference genome (hg19_random) using Burrows-Wheeler Aligner (version 0.6.2)^44^. Somatic single nucleotide mutations were called using both Strelka (version 1.0.7)^45^, and MuTect (version 1.1.4)^46^, while indels were called using only Strelka (version 1.0.7). ANNOVAR^47^ was used to annotate all the final mutation calls. Somatic copy number variation was assessed using CELLULOID^11^. Data generated in this work may be accessed using our repository portal (https://cbioportal.ca/cbioportal/study/summary?id=pancreas_pdx_2021). Sanger sequencing was performed to verify *KRAS*, *EGFR* and *BRAF* mutations status using primer pairs as listed in Supplementary Table S9.

Copy number alterations were profiled using Illumina Infinium Genotyping Kit (Illumina Inc.). Genomic DNA (500 ng) samples were amplified, fragmented, precipitated and hybridized to Illumina Human Omni 2.5 M SNP chip array as specified by manufacture. Intensity files were converted for quantification and normalization in GenomeStudio using HumanOmni2.5-8v1_A.bpm manifest and cluster files from Hg19. Copy gains and losses were detected using ASCAT algorithm^48^. ASCAT utilizes the Piecewise Constant Fitting (PCF) method to define segments and assigns a copy number for each allele. Hence, the total copy number of a segment was derived from the sum of the allele specific copy numbers. Copy gains and losses were defined as having 0.5 copy above (for gain) and 0.5 copy below (for loss) relative to ASCAT estimated sample ploidy. Hierarchical clustering was performed on segmented copy number Log R values using Pearson dissimilarity (1-Pearson correlation coefficient).

Gene expression profiles were generated from RNA (200 ng) of flash frozen xenograft tumors using the Illumina Whole Genome Gene Expression DASL Assay, using manufacturer’s protocol for HT-12 V4 BeadChip (Illumina Inc.), and the fluorescence data files were converted for quantification using GenomeStudio® software without normalization or background subtraction. Quality assessment, pre-processing and normalization of the raw expression data were implemented using R/Bioconductor package *lumi*^49^. Raw data was log2-transformed, back-ground subtracted, and normalized using a variance-stabilizing transformation algorithm. Correlation and fold-change analysis were done on probes detected (p < 0.01) in at least two passages within a series. Pearson dissimilarity (1-Pearson correlation coefficient) was used to perform hierarchical clustering on the expression values of samples with multiple passages.

### Generation of XDO models

Xenograft-derived organoids (XDOs) were established from cryopreserved PDX tumor fragments (< 5 mm^3^). Thawed fragments were dissociated using 100 g/ml Liberase (TH research grade, Catalog #5401135001, Roche Molecular Sysems, Inc.) for 90 min at 37 °C, then further with Gibco TrypLE Express (Catalog #12,605,036, ThermoFisher Scientific Inc.) for 10–20 min at 37 °C, based on the method described by Fujii et al.^50^. Dissociated cells were collected and embedded in growth factor-reduced Matrigel™ (Catalog #354,230, Corning Inc.), and overlaid with hPDAC growth medium^51^. Standard PMLB protocols were followed to propagate models for a minimum of 5 passages to be considered stable long-term cultures, and quality assurance was performed to evaluate concordance of organoid with matched PDX and patient histology and DNA fingerprinting.

### Drug testing on pancreatic tumor models in vitro and in vivo

An in vitro drug screen was conducted on organoids. Dissociated cells were seeded on top of a thin layer of Matrigel™ in culture medium in a 384-well plate (3,000 cells per well)^52^. All pharmaceutical agents were added the next day, in a 6-point concentration series from 10 uM to 0.001 uM to wells in triplicate. Cell viability was assessed by ATP quantification using the CellTiter-Glo 3D luminescence-based assay (Catalog #G9681, Promega Corp.). Viability values were normalized to vehicle control wells and dose concentrations were log10-transformed. Drug dose response scores were calculated using area under the curve (AUC) values for each drug, and were normalized by dividing the AUC value by the maximum area for the concentration range measured for each drug. CompuSyn software^44^ was used to calculate combination indices for combination drug studies.

PDX drug screen was performed using banked cryopreserved PDX fragments that were thawed and implanted in donor mice, tumors were harvested at full size, and fragments were generated and implanted in male mouse replicates for drug treatments. When tumor replicates averaged 100 mm^3^, mice were randomized (n = 5/group) for treatment with vehicle (0.5% hydroxyethyl-cellulose, 0.2% Tween 80, 99.3% distilled water), or MEK1/2 inhibitor trametinib (1 mg/kg, oral gavage, daily), or pan-BRAF inhibitor LY3009120 (35 mg/kg, oral gavage, daily). Tumor sizes and mouse body weights were measured twice weekly without knowledge of treatment group assignments. Research grade drugs were purchased from UHN-Shanghai Research & Development Co., Ltd (Shanghai, China).

### Statistical analysis

Patient outcome endpoints were characterized by survival time, and time to relapse with respect to diagnosis date. Survival was defined as time from diagnosis to death or last follow-up. Any death was considered an event. The probability of survival was estimated using Kaplan–Meier method. The Cox proportional hazards model was used to estimate the hazards ratio for the adjusted effect. The p-values cited in the multivariate analysis were based on the Wald test within the Cox proportional hazards model.

The difference in survival distribution between different groups was tested using log-rank test. The relapse was considered as an event and death without relapse as competing risks. The probability of relapse was calculated using the cumulative incidence approach. The difference between the distributions of time to relapse between groups was calculated using the Gray test.

PDX growth rates were estimated using log-linear mixed effects models. The models include fixed effects of week, treatment, PDX and all interactions as well as a random intercept and day effect. This type of models allows the test the difference in the treatment effect across the models used. Tumor volumes and latencies of PDXs were log transformed to stabilize the variance.

## Supplementary Information

Supplementary Information 1.

Supplementary Information 2.

## Data Availability

Our PMLB PDX models are listed in the open global catalogue of PDX models at the PDXFinder repository (pdxfinder.org). Somatic mutations data generated in this work may be accessed using the CBIOPORTAL.CA repository (https://cbioportal.ca/cbioportal/study/summary?id=pancreas_pdx_2021).

## References

[1] Howlader N, Noone AM, Krapcho M, Miller D, Brest A, Yu M (2019). SEER Cancer Statistics Review 1975–2016.

[2] Klein F, Jacob D, Bahra M, Pelzer U, Puhl G, Krannich A (2014). Prognostic factors for long-term survival in patients with ampullary carcinoma: The results of a 15-year observation period after pancreaticoduodenectomy. HPB Surg..

[3] Demeure MJ, Craig DW, Sinari S, Moses TM, Christoforides A, Dinh J (2012). Cancer of the ampulla of Vater: Analysis of the whole genome sequence exposes a potential therapeutic vulnerability. Genome Med..

[4] Sandhu V, Wedge DC, Bowitz Lothe IM, Labori KJ, Dentro SC, Buanes T (2016). The genomic landscape of pancreatic and periampullary adenocarcinoma. Cancer Res..

[5] Wardell CP, Fujita M, Yamada T, Simbolo M, Fassan M, Karlic R (2018). Genomic characterization of biliary tract cancers identifies driver genes and predisposing mutations. J. Hepatol..

[6] Huguet JM, Lobo M, Labrador JM, Boix C, Albert C, Ferrer-Barcelo L (2019). Diagnostic-therapeutic management of bile duct cancer. World J. Clin. Cases.

[7] Pea A, Riva G, Bernasconi R, Sereni E, Lawlor RT, Scarpa A (2018). Ampulla of Vater carcinoma: Molecular landscape and clinical implications. World J. Gastrointest. Oncol..

[8] Lai E, Puzzoni M, Ziranu P, Pretta A, Impera V, Mariani S (2019). New therapeutic targets in pancreatic cancer. Cancer Treat. Rev..

[9] Nevala-Plagemann C, Hidalgo M, Garrido-Laguna I (2020). From state-of-the-art treatments to novel therapies for advanced-stage pancreatic cancer. Nat. Rev. Clin. Oncol..

[10] Bailey P, Chang DK, Nones K, Johns AL, Patch AM, Gingras MC (2016). Genomic analyses identify molecular subtypes of pancreatic cancer. Nature.

[11] Notta F, Chan-Seng-Yue M, Lemire M, Li Y, Wilson GW, Connor AA (2016). A renewed model of pancreatic cancer evolution based on genomic rearrangement patterns. Nature.

[12] Aguirre AJ, Nowak JA, Camarda ND, Moffitt RA, Ghazani AA, Hazar-Rethinam M (2018). Real-time genomic characterization of advanced pancreatic cancer to enable precision medicine. Cancer Discov..

[13] Cancer Genome Atlas Research Network. Electronic address aadhe, Cancer Genome Atlas Research N (2017). Integrated genomic characterization of pancreatic ductal adenocarcinoma. Cancer Cell.

[14] Jones MR, Williamson LM, Topham JT, Lee MKC, Goytain A, Ho J (2019). NRG1 gene fusions are recurrent, clinically actionable gene rearrangements in KRAS wild-type pancreatic ductal adenocarcinoma. Clin. Cancer Res..

[15] Heining C, Horak P, Uhrig S, Codo PL, Klink B, Hutter B (2018). NRG1 fusions in KRAS wild-type pancreatic cancer. Cancer Discov..

[16] Li X, Zhao H, Gu J, Zheng L (2016). Prognostic role of HER2 amplification based on fluorescence in situ hybridization (FISH) in pancreatic ductal adenocarcinoma (PDAC): A meta-analysis. World J. Surg. Oncol..

[17] Adamska A, Domenichini A, Falasca M (2017). Pancreatic ductal adenocarcinoma: Current and evolving therapies. Int. J. Mol. Sci..

[18] Gao H, Korn JM, Ferretti S, Monahan JE, Wang Y, Singh M (2015). High-throughput screening using patient-derived tumor xenografts to predict clinical trial drug response. Nat. Med..

[19] Golan T, Stossel C, Schvimer M, Atias D, Halperin S, Buzhor E (2017). Pancreatic cancer ascites xenograft-an expeditious model mirroring advanced therapeutic resistant disease. Oncotarget.

[20] Gendoo DMA, Denroche RE, Zhang A, Radulovich N, Jang GH, Lemire M (2019). Whole genomes define concordance of matched primary, xenograft, and organoid models of pancreas cancer. PLoS Comput. Biol..

[21] Martinez-Garcia R, Juan D, Rausell A, Munoz M, Banos N, Menendez C (2014). Transcriptional dissection of pancreatic tumors engrafted in mice. Genome Med..

[22] Mattie M, Christensen A, Chang MS, Yeh W, Said S, Shostak Y (2013). Molecular characterization of patient-derived human pancreatic tumor xenograft models for preclinical and translational development of cancer therapeutics. Neoplasia.

[23] Wennerstrom AB, Lothe IM, Sandhu V, Kure EH, Myklebost O, Munthe E (2014). Generation and characterisation of novel pancreatic adenocarcinoma xenograft models and corresponding primary cell lines. PLoS ONE.

[24] Xie T, Musteanu M, Lopez-Casas PP, Shields DJ, Olson P, Rejto PA (2015). Whole exome sequencing of rapid autopsy tumors and xenograft models reveals possible driver mutations underlying tumor progression. PLoS ONE.

[25] Cavalloni G, Peraldo-Neia C, Sassi F, Chiorino G, Sarotto I, Aglietta M (2016). Establishment of a patient-derived intrahepatic cholangiocarcinoma xenograft model with KRAS mutation. BMC Cancer.

[26] Vaeteewoottacharn K, Pairojkul C, Kariya R, Muisuk K, Imtawil K, Chamgramol Y (2019). Establishment of highly transplantable cholangiocarcinoma cell lines from a patient-derived xenograft mouse model. Cells.

[27] Hou S, Tiriac H, Sridharan BP, Scampavia L, Madoux F, Seldin J (2018). Advanced development of primary pancreatic organoid tumor models for high-throughput phenotypic drug screening. SLAS Discov..

[28] Jones S, Zhang X, Parsons DW, Lin JC, Leary RJ, Angenendt P (2008). Core signaling pathways in human pancreatic cancers revealed by global genomic analyses. Science.

[29] Biankin AV, Waddell N, Kassahn KS, Gingras MC, Muthuswamy LB, Johns AL (2012). Pancreatic cancer genomes reveal aberrations in axon guidance pathway genes. Nature.

[30] Pergolini I, Morales-Oyarvide V, Mino-Kenudson M, Honselmann KC, Rosenbaum MW, Nahar S (2017). Tumor engraftment in patient-derived xenografts of pancreatic ductal adenocarcinoma is associated with adverse clinicopathological features and poor survival. PLoS ONE.

[31] Garcia PL, Council LN, Christein JD, Arnoletti JP, Heslin MJ, Gamblin TL (2013). Development and histopathological characterization of tumorgraft models of pancreatic ductal adenocarcinoma. PLoS ONE.

[32] Loukopoulos P, Kanetaka K, Takamura M, Shibata T, Sakamoto M, Hirohashi S (2004). Orthotopic transplantation models of pancreatic adenocarcinoma derived from cell lines and primary tumors and displaying varying metastatic activity. Pancreas.

[33] Perez-Torras S, Vidal-Pla A, Miquel R, Almendro V, Fernandez-Cruz L, Navarro S (2011). Characterization of human pancreatic orthotopic tumor xenografts suitable for drug screening. Cell. Oncol. (Dordr).

[34] John T, Kohler D, Pintilie M, Yanagawa N, Pham NA, Li M (2011). The ability to form primary tumor xenografts is predictive of increased risk of disease recurrence in early-stage non-small cell lung cancer. Clin. Cancer Res..

[35] Karamboulas C, Bruce JP, Hope AJ, Meens J, Huang SH, Erdmann N (2018). Patient-derived xenografts for prognostication and personalized treatment for head and neck squamous cell carcinoma. Cell. Rep..

[36] Leiting JL, Murphy SJ, Bergquist JR, Hernandez MC, Ivanics T, Abdelrahman AM (2020). Biliary tract cancer patient-derived xenografts: Surgeon impact on individualized medicine. JHEP Rep..

[37] Chen SH, Zhang Y, Van Horn RD, Yin T, Buchanan S, Yadav V (2016). Oncogenic BRAF deletions that function as homodimers and are sensitive to inhibition by RAF dimer inhibitor LY3009120. Cancer Discov..

[38] Ng SS, Tsao MS, Nicklee T, Hedley DW (2001). Wortmannin inhibits pkb/akt phosphorylation and promotes gemcitabine antitumor activity in orthotopic human pancreatic cancer xenografts in immunodeficient mice. Clin. Cancer Res..

[39] Kilkenny C, Browne WJ, Cuthill IC, Emerson M, Altman DG (2010). Improving bioscience research reporting: The ARRIVE guidelines for reporting animal research. PLoS Biol..

[40] Conte N, Mason JC, Halmagyi C, Neuhauser S, Mosaku A, Yordanova G (2019). PDX Finder: A portal for patient-derived tumor xenograft model discovery. Nucleic Acids Res..

[41] Shi R, Radulovich N, Ng C, Liu N, Notsuda H, Cabanero M (2020). Organoid cultures as preclinical models of non-small cell lung cancer. Clin. Cancer Res..

[42] Hudson TJ, Anderson W, Artez A, Barker AD, Bell C (2010). International network of cancer genome projects. Nature.

[43] Conway T, Wazny J, Bromage A, Tymms M, Sooraj D, Williams ED (2012). Xenome: A tool for classifying reads from xenograft samples. Bioinformatics.

[44] Li H, Durbin R (2009). Fast and accurate short read alignment with Burrows-Wheeler transform. Bioinformatics.

[45] Saunders CT, Wong WS, Swamy S, Becq J, Murray LJ, Cheetham RK (2012). Strelka: accurate somatic small-variant calling from sequenced tumor-normal sample pairs. Bioinformatics.

[46] Cibulskis K, Lawrence MS, Carter SL, Sivachenko A, Jaffe D, Sougnez C (2013). Sensitive detection of somatic point mutations in impure and heterogeneous cancer samples. Nat. Biotechnol..

[47] Wang K, Li M, Hakonarson H (2010). ANNOVAR: functional annotation of genetic variants from high-throughput sequencing data. Nucleic Acids Res..

[48] Van Loo P, Nordgard SH, Lingjaerde OC, Russnes HG, Rye IH, Sun W (2010). Allele-specific copy number analysis of tumors. Proc. Natl. Acad. Sci. USA.

[49] Du P, Kibbe WA, Lin SM (2008). lumi: a pipeline for processing Illumina microarray. Bioinformatics.

[50] Fujii M, Shimokawa M, Date S, Takano A, Matano M, Nanki K (2016). A colorectal tumor organoid library demonstrates progressive loss of niche factor requirements during tumorigenesis. Cell Stem Cell.

[51] Boj SF, Hwang CI, Baker LA, Chio II, Engle DD, Corbo V (2015). Organoid models of human and mouse ductal pancreatic cancer. Cell.

[52] van de Wetering M, Francies HE, Francis JM, Bounova G, Iorio F, Pronk A (2015). Prospective derivation of a living organoid biobank of colorectal cancer patients. Cell.

