# Supplementary material for: Patient-derived tumor xenograft and organoid models established from resected pancreatic, duodenal and biliary cancers
Source: Sci Rep. 2021 May 19;11:10619. doi: 10.1038/s41598-021-90049-1 (PMC8134568; doi:10.1038/s41598-021-90049-1)
Supplement: Supplementary file 1 — Supplementary Information 1. [file 41598_2021_90049_MOESM1_ESM.pdf]

Supplemental Data

for

Patient-derived Tumor Xenograft and Organoid Models Established from Resected Pancreatic, Duodenal and Biliary Cancers

Nhu-An Pham, Nikolina Radulovich, Emin Ibrahimov, Sebastiao N. Martins- Filho, Quan Li, Melania Pintilie, Jessica Weiss, Vibha Raghavan, Michael Cabanero, Robert E. Denroche, Julie M. Wilson, Cristiane Metran-Nascente, Ayelet Borgida, Shawn Hutchinson, Anna Dodd, Michael Begora, Dianne Chadwick, Stefano Serra, Jennifer J Knox, Steven Gallinger, David W Hedley, Lakshmi Muthuswamy, Ming-Sound Tsao

Supplementary Figures are listed first followed by Supplementary Tables.

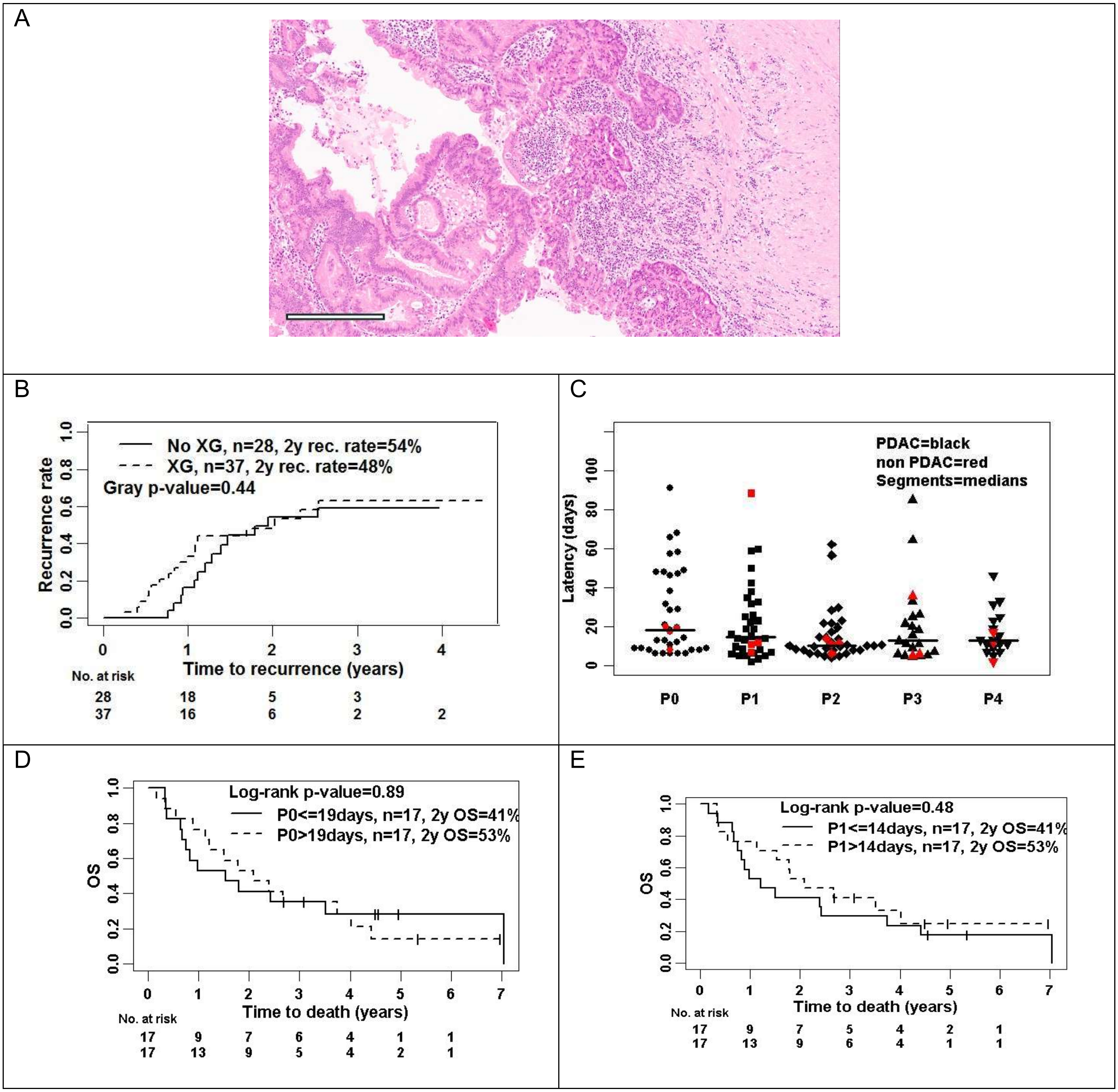
A
B
No XG, n=28, 2y rec. rate=54%
XG, n=37, 2y rec. rate=48%
Gray p-value=0.44
Recurrence rate
Time to recurrence (years)
No. at risk
28 18 5 3
37 16 6 2 2
C
PDAC=black
non PDAC=red
Segments=medians
Latency (days)
P0 P1 P2 P3 P4
D
Log-rank p-value=0.89
P0<=19days, n=17, 2y OS=41%
P0>19days, n=17, 2y OS=53%
OS
Time to death (years)
No. at risk
17 9 7 6 4 1 1
17 13 9 5 4 2 1
E
Log-rank p-value=0.48
P1<=14days, n=17, 2y OS=41%
P1>14days, n=17, 2y OS=53%
OS
Time to death (years)
No. at risk
17 9 7 5 4 2 1
17 13 9 6 4 1 1

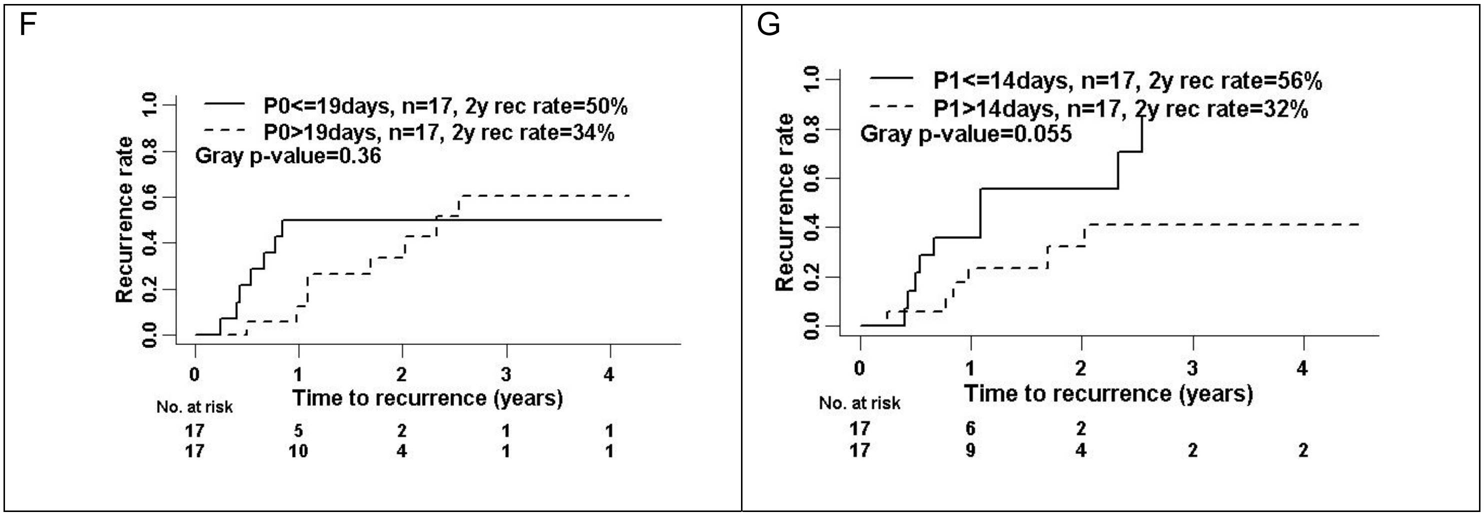

Supplementary Figure S1. Engraftment success and corresponding patients' clinical outcome. (A) An intraductal papillary mucinous neoplasm (IPMN) patient tumor (OCIP250) showed focal invasion in the H&E stained image. Scale bar is 300 microns. (B) There was no significant difference in recurrence of patients with their corresponding engraftment successes (XG) and failures (No XG). (C) Model latency time from implant to first detection by palpitation in mouse replicates was monitored in a subset of PDAC (black symbols), and other pancreas tumor types (red symbols). Latency durations (P0/P1) in PDAC models were correlated with overall survival (D-E) or with recurrence rate (F-G).

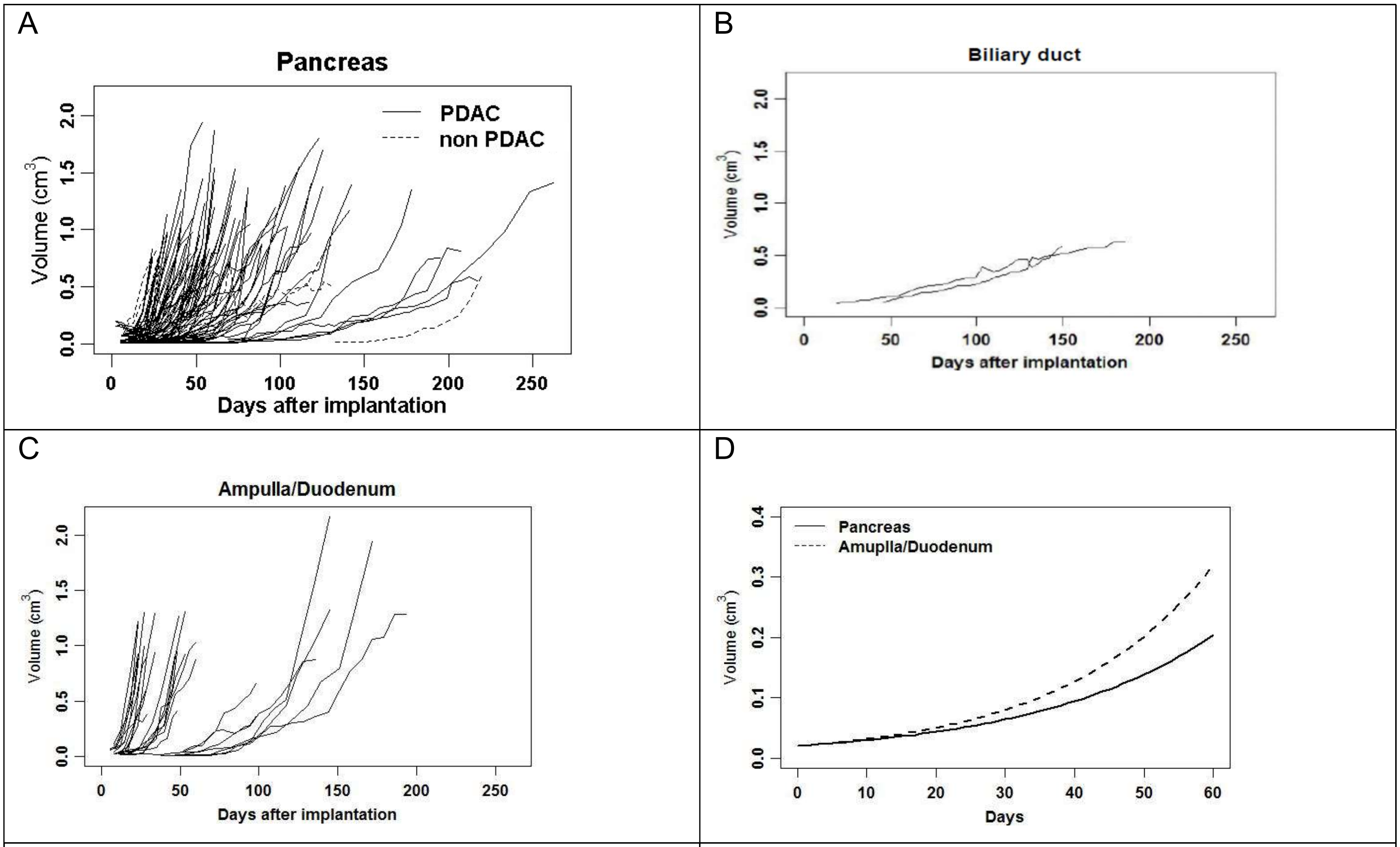

E

Primary site	The rate of growth per day (log scale)	P-value
pancreas	0.038	
duodenum	0.046	<0.0001

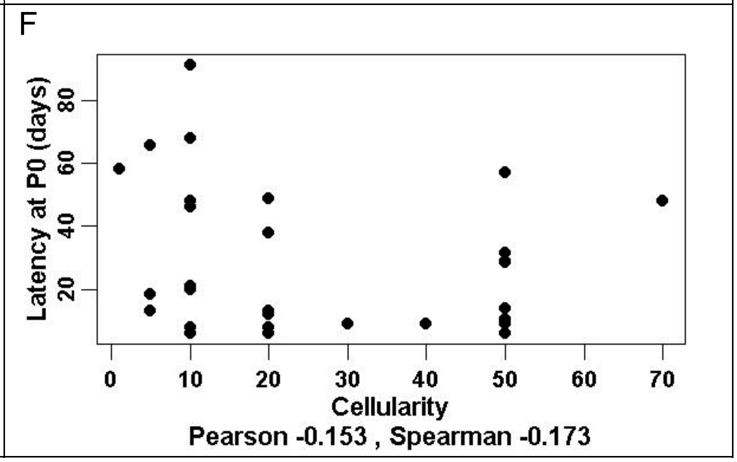

Supplementary Figure S2. Growth characteristics of PDX. Model growth rates for (A) PDAC and non-PDAC pancreas types, (B) biliary duct, and (C) duodenum cancers. (D) Growth rates were log transformed and the predicted curves representing pancreas and duodenum models, based on a mixed effect modelling, with significant growth rate differences between these two types (P-value <0.0001), (E). Since there was only data on 2 biliary duct models this category was excluded from the mixed effect modelling. (F) Latency duration from time of implant of patient tumor resected fragment (P0) to first palpable detection was compared to percent of tumor cellularity of tissue fragment adjacent to implanted fragment.

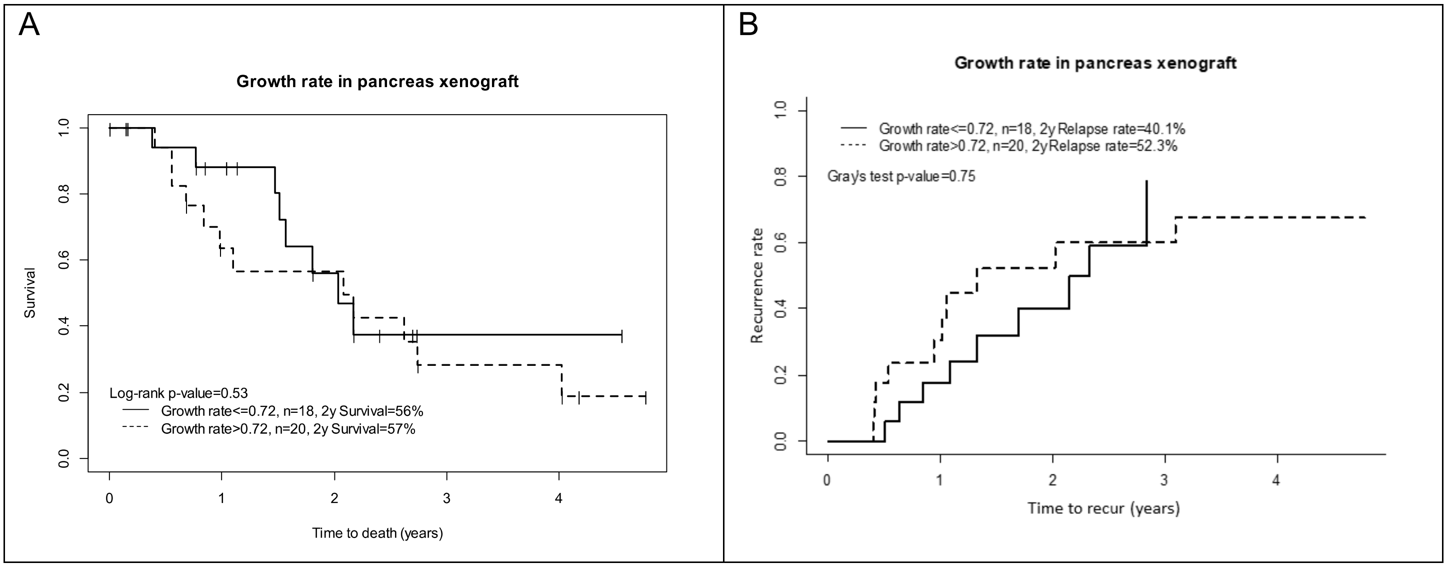

Supplementary Figure S3. Kaplan-Meier curves as evaluated by growth rates of PDAC models, (A) overall survival years, or (B) recurrence (dichotomized at median slope).

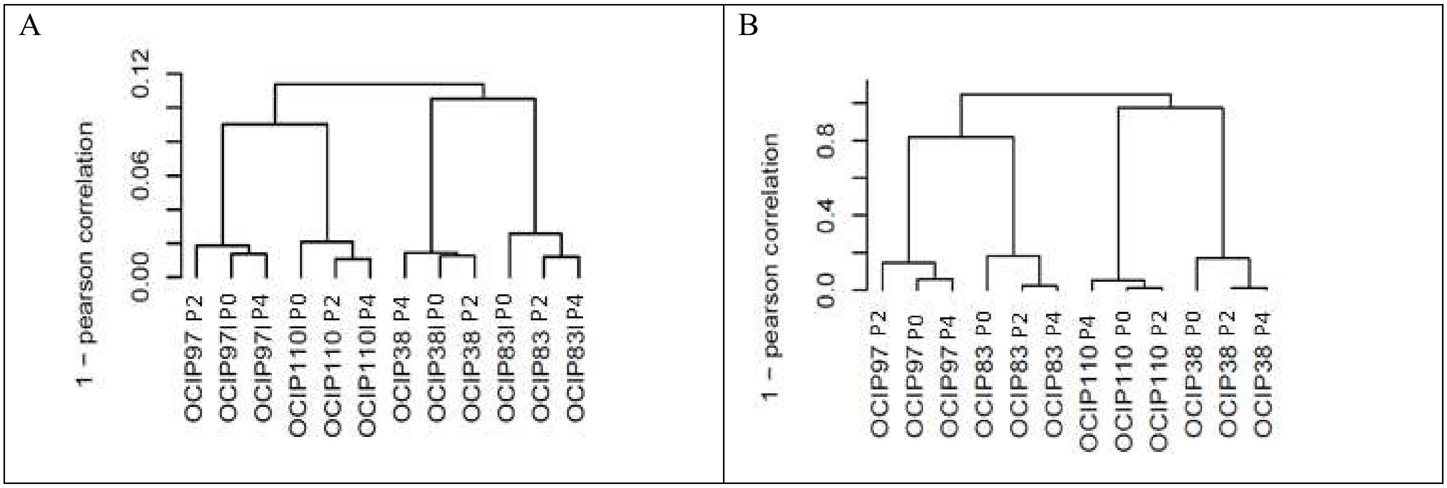

Supplementary Figure S4. Genomic stability in four PDAC xenograft models as depicted by hierarchical clustering of (A) copy number variants, and (B) gene expression profiles.

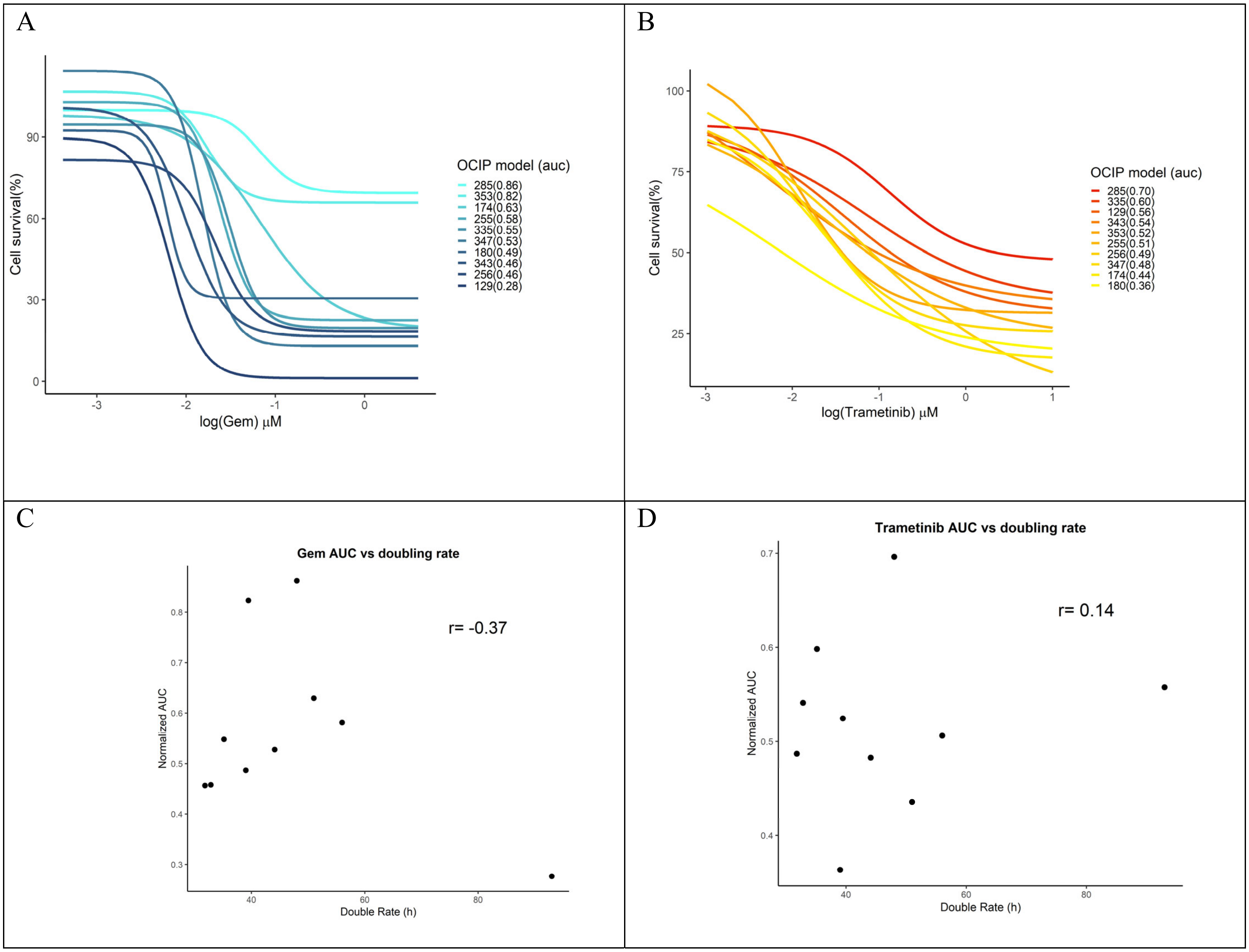

Supplementary Figure S5. Organoid drug dose responses. Cell survival was measured post-treatment with (A) gemcitabine and (B) trametinib. Scatter plots of organoid drug sensitivity scores for (C) gemcitabine and (D) trametinib were compared to XDO doubling rate, with correlation coefficient r values calculated, respectively.

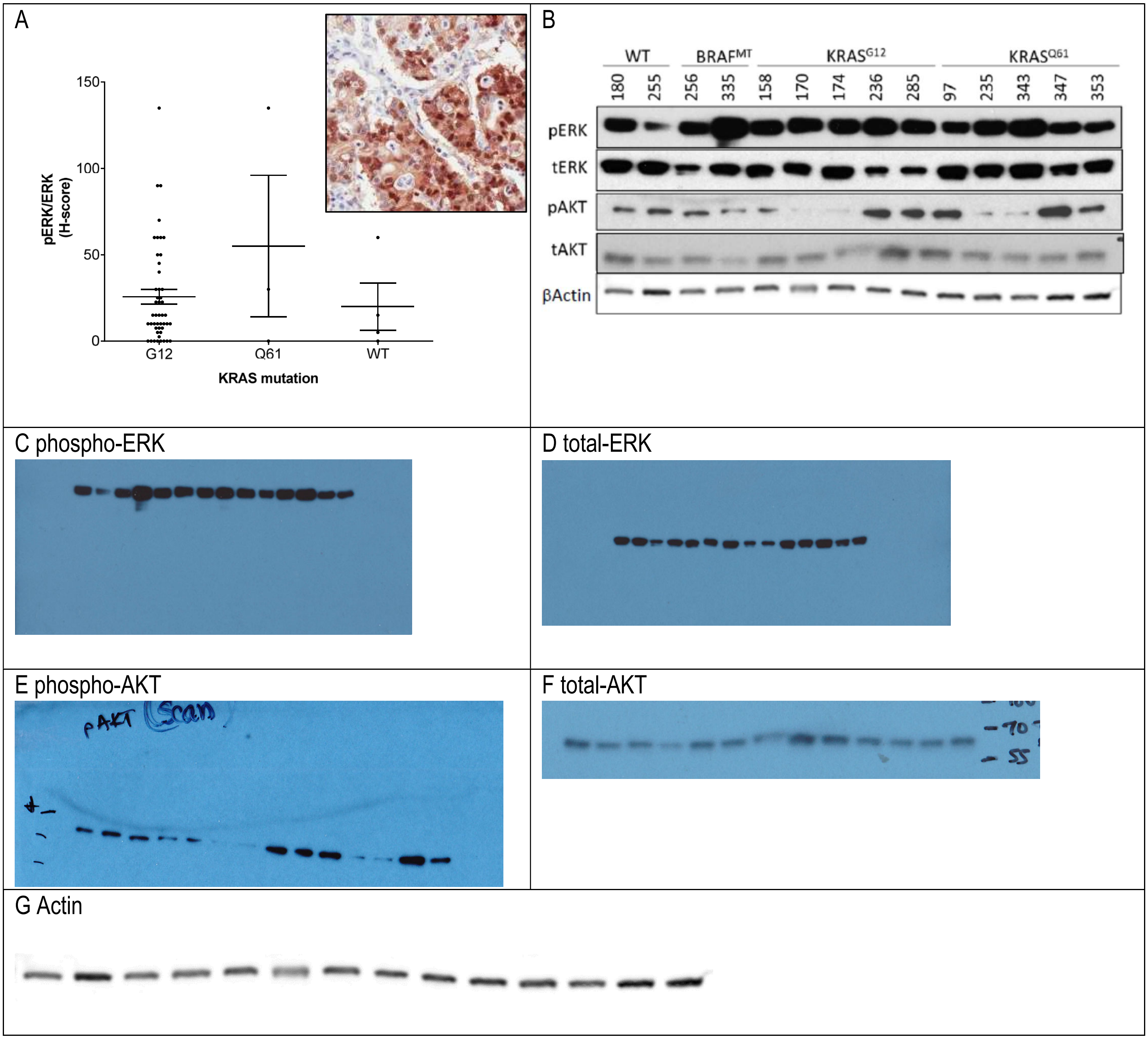

Supplementary Figure S6. Pathway activation characterized by phosphorylated-ERK (pERK) and pAKT in PDX models. Two models (OCIP235 and 236 are extraneous to this manuscript). (A) Immunohistochemistry evaluations were performed and H-scores (% cells x staining intensity [0-3]) were plotted. (B) PDX models were characterized by their phospho-proteins (pERK and pAKT) compared to their respective total protein (tERK, tAKT) and α-tubulin levels. This image is a composite of original scans in color or black and white converted (C-G).

A) OCIP285 (*KRAS* Q61R)

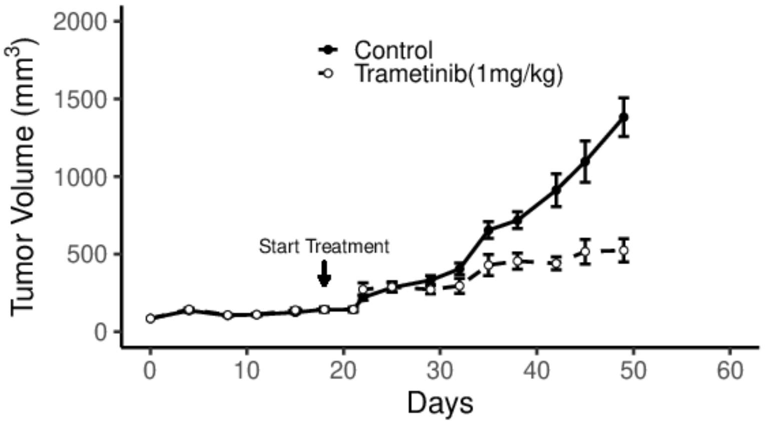

B) OCIP180 (triple negative wild-type *KRAS/EGFR/BRAF*)

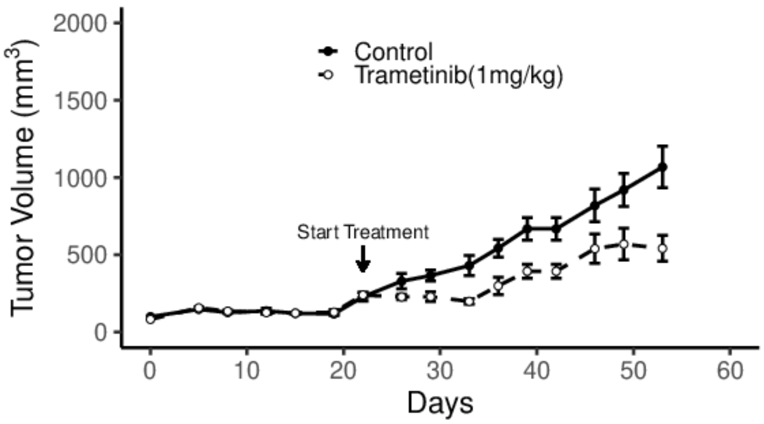

C) Growth rates

Controls		Trametinib	
OCIP180	OCIP285	OCIP180	OCIP285
1.40	1.70	1.28	1.32

Supplementary Figure S7. Trametinib anti-tumor effects on *KRAS* mutant and wild-type PDX models. (A) Growth rate of OCIP285 was slower when treated with trametinib compared to vehicle control (0.78, P<0.001). (B) Similarly, OCIP180 showed a slower growth rate with trametinib treatment compared to vehicle control, (0.91, P<0.001). Mean +/- SD were indicated for growth curves of vehicle control and trametinib groups (n=5 tumors per group). (C) Estimated growth rates per week were calculated using log-linear mixed effects models for the PDXs. The models include fixed effects of week, treatment, PDX and all interactions as well as a random intercept and day effect. This type of modelling tested the difference in the treatment effect across these PDXs.

A Phospho-ERK

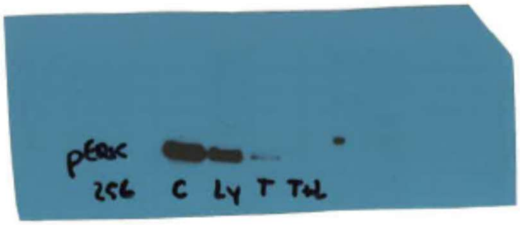

B Phospho-AKT

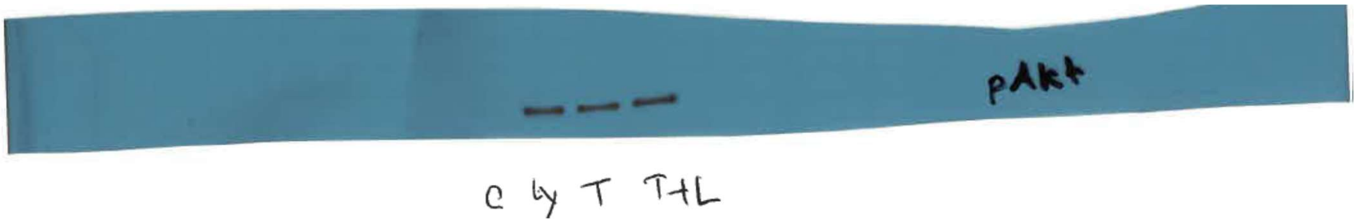

C Total-ERK and Total-AKT (background incomplete stripping of beta-actin signal). First 4 bands belong to OCIP256

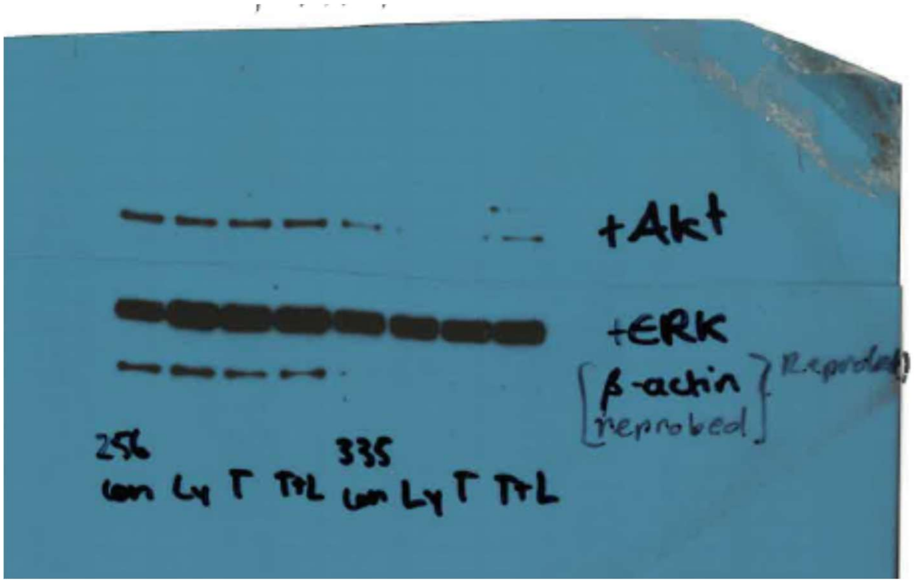

D β-actin (first 4 bands specific to OCIP256)

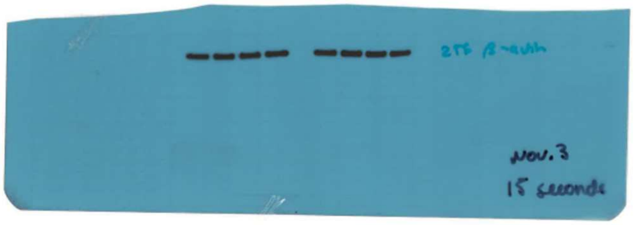

Supplementary Figure S8. Organoid OCIP256 treated with LY3009120 (LY) and/or trametinib (Tram). Protein lysates were analysed by Western blots and scanned films (A-D) show relative expression levels of phospho-ERK (pERK) and phospho-AKT (pAKT) and their respective total protein levels.

Supplementary Table S1. Outcome and genomics data summary

Cases	PDAC	other pancreas	biliary duct	duodenum / ampulla	Total
**Total**	169	30	48	29	276
**No XG**	64	17	31	4	116 (42%)
**outcome**	46	-	-	-	
**XG at P0**	105	13	17	25	160 (58%)
**Failed to expand >P0**	16	3	9	5	33
**Outcome**	13	-	-	-	
**SXG**	89	10	8	20	127 (46%)
**outcome**	79	-	-	-	
**whole genome**	8	-	-	-	
**whole exome**	23	-	-	-	
**CNV**	19	-	-	-	

Number of tumor specimens collected for xenograft model generation. Initial engraftment of patient tumor specimens in NOD SCID mice, engraftment (XG) at passage 0 (P0), and followed by serial mouse propagation for another two passages to be considered stable engraftment (SXG). Subsets of the pancreatic ductal adenocarcinoma (PDAC) models have follow-up patient data and genomics data including copy number variants (CNV).

Supplementary Table S2. See Excel table for list of xenograft models and their characteristics.

Supplementary Table S3. Impact of implant site and PDAC engraftment.

Total implanted	Pancreas ductal adenocarcinoma engraftment (P0)	
	Orthotopic	Flank
71	16 (22%)	71 (100%)

Both sites of xenograft implant, subcutaneous and orthotopic, were attempted for a subset of PDAC cases in Table 1 when specimen size was sufficient.

Supplementary Table S4. Pancreatic ductal adenocarcinoma patient characteristics and model engraftment.

Variable	Categories	Total	noXG	SXG	*p* value
**Age**	Median (range)	65 (37-85)	63 (37-85)	65 (39-83)	0.98^
	unknown	29	19	10	-
**Sex**	F	85 (51%)	41 (51%)	44 (50%)	0.88*
	M	84 (49%)	39 (46%)	45 (54%)	-
**Stage**	I	13 (8%)	-	-	-
	II	135 (80%)	-	-	-
	III	8 (5%)	-	-	-
	IV	6 (3%)	-	-	-
	unknown (ascites)	7 (4%)	-	-	-
**stage groups**	I/II	148 (91%)	66 (89%)	82 (93%)	0.41*
	III/IV	14 (9%)	8 (11%)	6 (7%)	-
**neoadjuvant therapy§**	None	99 (59%)	40 (24%)	59 (35%)	0.63**
	chemo	11 (7%)	5 (3%)	6 (4%)	-
	chemo+radiation	10 (6%)	5 (3%)	5 (3%)	-
	unknown	48 (28%)	30 (18%)	18 (11%)	-
**tumor cellularity**	median(range)	30 (0-80)	10 (0-80)	20 (1-70)	0.26^
	unknown	115	59	56	-

Tests were performed to evaluate differences in categories of patients that failed engraftment (noXG) compared to those that formed stable xenograft models (SXG). * Fisher exact test. ** Fisher exact test was applied for treatment vs. no treatment. ^Mann-Whitney test. §These specimens were collected prior to the implementation of standard neoadjuvant therapy. The pre-operative treatment (broadly termed here as neoadjuvant) was offered to some patients, with non-surgical disease due to blood vessel involvement, with a combination of low dose gemcitabine and radiotherapy, and some patients then proceed to surgery.

Supplementary Table S5A. Time to detection of tumor xenograft growth.

Passage #	count	Latency days median (range)	Wilcoxon signed-rank P-value
P0	33	18 (6-91)	
P1	32	14 (2-60)	0.13
P2	32	11(4-62)	0.017
P3	19	11 (5-81)	0.16
P4	16	13 (5-46)	0.23

Matched-models mouse replicates were compared at each passage to P0.

Supplementary Table S5B. Detection of PDAC PDX growth derived from patient specimen.

Post-implant at subcutaneous flank (days)	Models initiated at P0 (%)
1-10	11 (31%)
11-20	4 (11%)
21-30	3 (9%)
31-40	4 (11%)
41-50	4 (11%)
51-60	3 (9%)
60-70	0
71-80	0
81-90	2 (6%)
91-100	2 (6%)
102 and 110	2 (6%)
Total	35

Each PDX model was established with 1-4 mouse replicates, the replicate with the longest latency to first detection of growth was selected for representation.

Supplementary Table S6. Somatic mutations fidelity in primary tumors compared to corresponding PDX.

OCIP ID	# Primary mutations	# PDX mutations	% Primary mutations present in PDX	% PDX mutations present in Primary
OCIP84	17	225	18%	1%
OCIP96	50	40	30%	38%
OCIP83	45	76	58%	34%
OCIP129	33	56	61%	36%
OCIP135	62	93	61%	41%
OCIP134	66	56	62%	73%
OCIP26	16	115	63%	9%
OCIP110	39	41	64%	61%
OCIP62	55	227	65%	16%
OCIP232	151	201	70%	53%
OCIP125	14	45	71%	22%
OCIP111	50	41	76%	93%
OCIP114	26	62	77%	32%
OCIP132	49	85	78%	45%
OCIP30	28	39	79%	56%
OCIP190	59	63	80%	75%
OCIP88	146	144	83%	84%
OCIP40	42	90	83%	39%
OCIP353	48	52	83%	77%
OCIP341	52	65	85%	68%
OCIP167	34	41	94%	78%
OCIP347	52	56	94%	88%
OCIP332	64	65	95%	94%

Somatic mutations were counted in the exome region for non-synonymous, missense, and frameshift indel.

Supplementary Table S7. BRCA1/2 germline and somatic DNA damage repair gene mutations.

OCIP ID	Gene	Source	Alteration type	Annotation	Chromosome location
28	BRCA2	germline frame shift delpathogenicchr13:32914437 GT>G	germline frame shift del	pathogenic	chr13:32914437 GT>G
62	BRCA2	somatic missense mutation possibly_damagingchr13:32971138 C>A	somatic missense mutation	possibly_damaging	chr13:32971138 C>A
88	BRCA2	germline frame shift delpathogenicchr13:32914437 GT>G	germline frame shift del	pathogenic	chr13:32914437 GT>G
84	BRCA2	somatic frame shift delpathogenicchr13:32914210 CT>-	somatic frame shift del	pathogenic	chr13:32914210 CT>-
		somatic frame shift del not availablechr13:32915070 A>-	somatic frame shift del	not available	chr13:32915070 A>-
217	BRCA1	germline snp stopgainpathogenicchr17:41234451 G>A	germline snp stopgain	pathogenic	chr17:41234451 G>A
232	BRCA2	germline frame shift delpathogenicchr13:32911657 TC>T	germline frame shift del	pathogenic	chr13:32911657 TC>T
		somatic nonsense mutation not availablechr13:32953608 G>A	somatic nonsense mutation	none	chr13:32953608 G>A
		somatic nonsense mutation not availablechr13:32953608 G>A	somatic nonsense mutation	none	chr13:32953608 G>A

Supplementary Table S8. Review of PDX collections for pancreatic, ampullary-duodenal and bile duct cancers.

Author, Year	PMID	Cancer type	Origin	Patient specimens	Mouse strain	P0 PDX (% engrafment)	Models (>P0 or not specified) (% engraftment)	Drug studies	Any molecular profiling
PDXfinder.org	none	PDAC, ampulary-duodenal, bile duct	primary	na	NSG, Athymic nude, NMRI Nude, NOD SCID		3	sub-set	available
Leiting JF, 2020	32181445	bile duct	primary / metastasis	77	NOD SCID	40 (53%)	unknown	not done	not done
Cavalloni G, 2016	26868125	bile duct	primary / recurrences	17	NOD SCID/Shi-SCID	1 (6%)	1 (6%)	not done	available
Vaeteewoottacharn K, 2019	31126020	bile duct	primary	16	Balb/c Rag-2-/-/ Jak3-/- mice	13 (81%)	12 (75%)	not done	not done
Pergolini I, 2017	28854237	PDAC	primary / metastasis	133	Nude	57 (43%)	unknown	not done	not done
Garcia PL, 2013	24194913	PDAC	primary	41	CB17-/- SCID	35 (85%	34 (83%)	not done	not done
Xu W, 2019	31217882	PDAC	primary	26	NOD SCID	10 (38%)	5(19%)	not done	not done
Golan T, 2017	28489577	PDAC	ascites	17	Nude	12 (71%)	12 (71%)	sub-set	available

Supplementary Table S9. Primers *KRAS, EGFR, BRAF*.

Mutation	Forward primer	Reverse primer
PCR		
KRAS 12/13	5’ CCCACCTATAATGGTGAATATCTTCAA 3’	5’ TGTTTCTCCCTTCTCAGGATTCC 3’
KRAS 61	5’ AATGGTGAATATCTTCAAATGA 3’	CCCTTCTCAGGATTCCTACA
BRAF V487 c.1457_1471del15	5’ AGCCTTAGAAAACAAATGGAGTTT 3’	5’ CAGCCATACCATATAACATTGCATA 3’
BRAF_D594F c.1457_1471del15	5’AATGCTTGCTCTGATAGGAAAATGA 3’	5’ TGACTTTCTAGTAACTCAGCAGCATCT 3’
EGFR L747-P753	5’ ATCGCTGGTAACATCCACCCAGAT 3’	5’ TTCAGAGCCATGGACCCCCACA 3’
Sequencing		
KRAS 12/13	5’ ACCTTATGTGTGACATGTTCTAATATAC 3’	5’ ATGGTCCTGCACCAGTAATATGC 3’
KRAS 61	5’ CCCACCTATAATGGTGAATATCTTCAA 3’	5’ TGTTTCTCCCTTCTCAGGATTCC 3’
BRAF V487 c.1457_1471del15	5’GATTTAATAATGGTATGGAGTTAGGG 3’	5’GCATACTACTTAAAAGAATGTGGTTAAAG 3’
BRAF_D594F c.1457_1471del15	5’TTGCTCTGATAGGAAATGA 3’	5’TTCTAGTAACTCAGCAGCATCT 3’
EGFR L747-P753	5’ GGGTGCATCGGCTGGTAACAT 3’	5’ AGGTGGGCCTGAGGTTCAG 3’
